# 2024 Thai guidelines on the treatment of hypertension

**DOI:** 10.2478/abm-2025-0034

**Published:** 2025-12-31

**Authors:** Sirisawat Kunanon, Praew Kotruchin, Pairoj Chattranukulchai, Chavalit Chotruangnapa, Weranuj Roubsanthisuk, Prin Vathesatogkit, Tada Kunavisarut, Songkwan Silaruks, Sirakarn Tejavanija, Tuangsit Wataganara, Piengbulan Yapan, Nijasri Suwanwela, Pongamorn Bunnag, Buncha Satirapoj, Surapun Sitthisook, Rapeephon Kunjara Na Ayudhya, Apichard Sukonthasarn

**Affiliations:** Division of Hypertension, Department of Medicine, Faculty of Medicine, Siriraj Hospital, Mahidol University, Bangkok 10700, Thailand sirisawat.wan@mahidol.ac.th; Department of Emergency Medicine, Faculty of Medicine, Khon Kaen University, Khon Kaen 42000, Thailand; Division of Cardiovascular Medicine, Department of Medicine, Faculty of Medicine, Chulalongkorn University, King Chulalongkorn Memorial Hospital, Bangkok 10300, Thailand; Division of Cardiology, Department of Medicine, Faculty of Medicine, Ramathibodi Hospital, Mahidol University, Bangkok 10400, Thailand; Division of Endocrinology and Metabolism, Department of Medicine, Faculty of Medicine, Siriraj Hospital, Mahidol University, Bangkok 10700, Thailand; Department of Medicine, Faculty of Medicine, Khon Kaen University, Khon Kaen 42000, Thailand; Department of Medicine, Phramongkutklao Hospital and College of Medicine, Bangkok 10400, Thailand; Department of Obstetrics and Gynecology, Faculty of Medicine, Siriraj Hospital, Mahidol University, Bangkok 10700, Thailand; Division of Neurology, Department of Medicine, Faculty of Medicine, Chulalongkorn University, King Chulalongkorn Memorial Hospital, Bangkok 10300, Thailand; Division of Endocrinology and Metabolism, Department of Medicine, Faculty of Medicine, Ramathibodi Hospital, Mahidol University, Bangkok 10400, Thailand; Vichaiyut Hospital and Medical Center, Bangkok 10400, Thailand; Thai Hypertension Society, Bangkok 10310, Thailand

**Keywords:** adult, guidelines, hypertension, Thailand

## Abstract

The committee of the 2024 Thai Guidelines on the Treatment of Hypertension has reviewed new developments in the body of knowledge, combined with expertise in real-life clinical practice and evidence collected from clinical studies worldwide. The Guidelines consist of newly highlighted key topics to be up to date and suitable for the country’s context. We still maintained the current office blood pressure (BP) cut-point of 140/90 mmHg for hypertension diagnosis. The new BP category, “BP at risk,” i.e., BP of 130–139/80–89 mmHg, was introduced. The out-of-office BP measurements, including home BP monitoring (HBPM) or ambulatory blood pressure monitoring (ABPM), are also advocated to confirm the diagnosis of hypertension. Target BP levels depend on the age of the patients i.e., 120–130/70–79 mmHg for patients age 18–65 years, 130–139/70–79 mmHg for patients over 65 years of age. There are 5 main groups of antihypertensive medication, that is, angiotensin-converting enzyme inhibitors, angiotensin receptor blockers, betablockers, calcium-channel blockers, and diuretics (thiazides and thiazide-like diuretics such as chlorthalidone and indapamide). Two types of medications should be started for most patients, except for frail elderly patients, patients with relatively low initial BP (140–149/90–99 mmHg), and low-risk patients; only 1 type of starting medication should be selected. Medication that is a combination of 2 types in 1 pill should be selected. Patient empowerment can be useful in sharing decisions in goal setting, provision of feedback channels, self-monitoring, education, and motivation, which the use of telemedicine and mobile health technologies can assist.

In 2012, the Thai Hypertension Society (THS) initiated a guideline for the management of hypertension in adults, which is the first hypertension guideline specifically used in Thai adults with high blood pressure (BP). This guideline was revised in 2015. THS published another updated Thai Guidelines on the Treatment of Hypertension in 2019. The significant disparities in the regional burden of hypertension among high–income and low- and middle–income regions are associated with low levels of awareness, treatment, and control rates in low–and middle–income countries [1]. The sixth National Health Examination Survey (NHES) in 2020 reported that 25.4% of Thai adults were hypertensive, which was higher than all the previous surveys. Furthermore, the percentage of awareness, treatment, and control of BP was all lower than in the previous reports [2]. Despite the effort of the Thai Ministry of Health in screening for individuals with high BP for many years, the percentage of awareness among people with high BP has not increased as expected.

To improve the management of hypertension in Thailand based on the best clinical evidence and healthcare resources, the THS has developed these 2024 Thai guidelines for the treatment of hypertension to be applied in adults aged 18 years and older with high BP, which is more suitable to be used in Thailand, which is among low–and middle–income Asian countries.

## Definition of the strength of recommendation and the quality of evidence

### Strength of recommendation

**Level I** means “Should be practiced” because the recommendation is highly reliable, beneficial, and worthwhile.

**Level IIa** means “Could be practiced” because the recommendation is moderately reliable, likely beneficial, and probably worthwhile.

**Level IIb** means “May be practiced” because the recommendation is not reliable enough, not sufficiently proven to be beneficial, and probably not worthwhile, but will not be harmful.

**Level III** means “Should not be practiced” or “Must not be practiced” because the recommendation is not beneficial and will probably be harmful.

### Quality of evidence

**A** means the evidence from various high-quality randomized controlled trials (RCTs) or meta-analyses.

**B** means the evidence from at least 1 high-quality RCT or a large-scale non-RCT with definitive outcome on advantages or disadvantages.

**C** means the evidence from other types of high-quality studies, a retrospective descriptive study, a registry, or experts’ opinions.

## Measurement of BP, definition, classification, and diagnosis of hypertension

### Measurement of BP

Accurate BP measurement is the fundamental process both in the diagnosis and management of hypertension. However, this important process is usually overlooked [3]. Standard BP measurement techniques are usually not followed by healthcare providers [4–6]. Three methods of BP measurement performed in clinical practice are office BP measurement, home or self-BP measurement, and ambulatory BP measurement. BP information from all methods of BP measurements is complementary. It will help clinicians to make the correct hypertension diagnosis and deliver suitable treatment for their patients, so all types of BP measurement should be used for the diagnosis and treatment of hypertension (*Strength of Recommendation I, Quality of Evidence A*).

#### BP measuring devices

To obtain patients’ reliable BP records, only validated BP measuring devices should be used (*Strength of Recommendation I, Quality of Evidence A*). Among the numerous brands and models of devices available worldwide, <10% have passed the international standard validation protocol [6]. The updated lists of validated devices can be found on certain websites, e.g., www.stridebp.org, www.bihsoc.org/bp-monitors, and www.validatebp.org. Periodic calibration of the device at least once a year is advisable for professional office and ambulatory BP devices. Still, it can be performed less frequently for home devices as long as the parts are maintained in good condition.

The arm cuff used with BP measuring devices must be of an appropriate size for the upper arm circumference of each subject. The bladder should encircle around 70.0%–80.0% of the subject’s mid-upper arm circumference for manual auscultatory devices. The selection of the appropriate arm cuff size according to the subject’s upper arm circumference is shown in Table 1. For automated electronic devices, many manufacturers currently provide the so-called “wide-range cuff,” which can fit the upper arms of most of the adult population. The wide-range cuff can be used in subjects with upper arm circumferences ranging from 22 cm to 42 cm. However, an extralarge arm cuff is sometimes needed for highly obese people.

**Table 1.** Appropriate arm cuff size according to subject’s upper arm circumference

Arm cuff size	Upper arm circumference (cm)	Bladder size in the arm cuff (cm)
Small	22–26	12 × 22
Medium (regular adult size)	27–34	16 × 30
Large	35–44	16 × 36
Extra-large	45–52	16 × 42

#### Preparation for BP measurements

**Figure 1.**
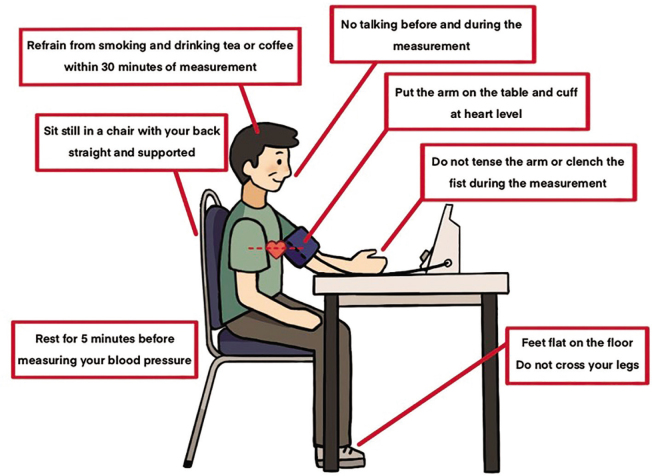
Subject preparation and standard position for BP measurement. BP, blood pressure.

Before BP measurements, the subject should be advised to avoid smoking, caffeine, and exercise for at least 30 min and should empty their bladder. The examination room should be quiet with a comfortable temperature and surroundings. The subject should sit on a chair with back support, upper arm bare, and rest on a table at heart level, legs uncrossed and feet flat on the floor (Figure 1). Subject should remain seated and relaxed for at least 3–5 min before measurements. Neither tightening nor gripping hands, and no talking, both before and between measurements.

### Office BP measurement

The guidelines in hypertension diagnosis and treatment are mainly derived from research using office BP measurements. There are 2 methods to obtain a subject’s BP in the office: the auscultatory method and the oscillometric method. The auscultatory method is traditional and serves as a reference standard for other methods. However, it requires well-trained personnel and consumes more time than the oscillometric method. These limitations have placed the oscillometric method into today’s clinical practice. The THS encourages healthcare personnel to be aware of the key steps in the auscultatory BP measurement technique, which is still the fundamental standard for all types of BP measurement.

Key steps in proper office BP measurement:

1. Wrap the proper size arm cuff over the subject’s upper arm 2–3 cm above the antecubital fossa to have the space for placing the stethoscope.
2. The marker on the arm cuff, which indicates the center of the bladder, should be on the brachial artery.
3. Estimate systolic blood pressure (SBP) by inflating the cuff while palpating the brachial pulse until brachial pulsation disappears. Then, release the cuff pressure slowly at around 2–3 mmHg/s until brachial pulsation resumes. SBP should be close to the pressure level, at which the arterial pulsation disappears and reappears.
4. Wait at least 1 min to have normal blood recirculation of that arm before the next measurement. Pulse rate and pulse rhythm can be determined while waiting.
5. Place the stethoscope over the brachial artery and raise the pressure to 20–30 mmHg over the estimated level of SBP from the palpation technique. Then, release the pressure slowly and listen to the Korotkoff sound, which will appear at intervals according to the heartbeat. The first sound heard is the Korotkoff sound phase 1; the pressure read at that time is the SBP. The BP at which the sound disappears, the Korotkoff sound phase 5, is the diastolic blood pressure (DBP).
6. Orthostatic hypotension can be screened in subjects with diabetes mellitus (DM), the elderly, or those with orthostatic symptoms (*Strength of Recommendation Ila, Quality of Evidence C*). Measure BP when the subject is in the lying position first, followed by measurement after standing up for 1 min and 3 min consecutively. Orthostatic hypotension is defined as a BP lowering ≥20/10 mmHg within 3 min after standing up from the supine position [7].
7. In subjects with arrhythmia, BP measurement with the auscultatory technique is preferred (*Strength of Recommendation IIa, Quality of Evidence C*). Using the average BP from multiple records is advised.

The oscillometric method has almost replaced this conventional method in clinical practice. After placing the arm cuff on the patient’s upper arm, press the start button. The device will inflate and deflate the cuff automatically. Arterial pulse waves will be detected and used for BP determination. SBP and DBP, together with pulse rate, will appear on the screen, usually within 1 min. In some models, multiple measurements can be taken to achieve average results. The oscillometric method can eliminate the terminal digit preference of healthcare providers, which is common in Thailand [4].

In the first office visit, BP should be measured simultaneously in both arms with an electronic device (*Strength of Recommendation I, Quality of Evidence A*). If an interarm SBP difference is >10 mmHg and confirmed with repeated measurements, the arm with the higher BP should be used for subsequent evaluation. If an interarm SBP difference is persistently between 15 mmHg and 20 mmHg, further investigations to confirm atherosclerosis or arterial diseases of the upper extremities are recommended. To avoid the white-coat effect, automated office blood pressure (AOBP) measurements can be done automatically in a quiet room without medical staff. This method is called unattended AOBP measurement. However, it is not possible in most hospitals in Thailand, and its BP threshold and target require more clinical outcome studies. THS strongly recommends standardized steps in office BP measurement for diagnosis and treatment of hypertension (*Strength of Recommendation I, Quality of Evidence A*).

### Home BP measurement or monitoring

Home BP measurement (HBPM) can confirm or exclude white-coat effect (higher office than out-of-office BP) and detect masked hypertension (masked hypertension is defined as normal office BP and high out-of-office BP), leading to a more precise diagnosis of BP phenotypes in hypertension. In pharmacologically treated cases, HBPM will enhance medication adherence of the patients, leading to better BP control. We, therefore, recommend using HBPM in all treated hypertensive cases (*Strength of Recommendation I, Quality of Evidence A*) unless the patient is incapable of performing it. Only validated oscillometric upper arm devices are recommended for home use (*Strength of Recommendation I, Quality of Evidence A*). Wrist or finger devices are not generally endorsed, but they may be required in markedly obese patients in whom BP measurement in the upper arm is too difficult to achieve or may be unreliable. Cuffless devices are still not standardized and cannot be advised for clinical use at this time (*Strength of Recommendation III, Quality of Evidence C*). Patients and/or their caregivers should learn about specific advice for the appropriate technique in using HBPM and how to record their BP properly (Table 2).

**Table 2.** Recommended frequency for HBPM

Conditions	Frequency of measurements
For hypertension diagnosis	Measure BP for 7 consecutive days
	In urgent conditions, it can be done for at least 3 consecutive days.
For treatment monitoring during antihypertensive medication adjustment	Measure BP for 7 consecutive days beginning 2 weeks after initiation or after changes in the treatment regimen and Measure BP at least 3 consecutive days during the week before a clinic visit.
For long-term follow-up in stable cases	Measure BP once or twice per week or Measure BP 7 d before each clinic visit, and at least over 1 week within 3 months of visit intervals.

1 BP, blood pressure; HBPM, home blood pressure measurement.

HBPM should be done twice a day, in the morning and the evening. Morning measurement should be performed within 1 h after waking up, after urination, before taking the morning dose of antihypertensive drugs, and before breakfast. Evening measurements should be made before retiring to sleep. A physician may adjust the frequency and time for BP measurement as needed in specific circumstances. At least 2 measurements should be made on each occasion, 1 min apart between readings. All readings should be recorded without selection bias. Subjects should always show their BP records to their healthcare provider at the clinic visit. Home BP is evaluated by using the mean values of all measurements, both in the morning and evening. When HBPM activates anxiety or causes stress to the subjects, it should be stopped. More details on HBPM can be found in the 2022 Thai Hypertension Society guidelines on HBPM [8].

### Ambulatory BP measurement or monitoring

**Figure 2.**
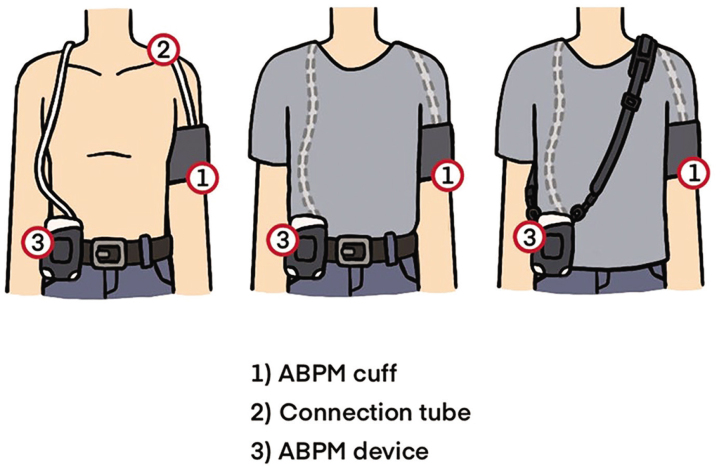
The fitting of ABPM device on patient’s upper arm. ABPM, ambulatory blood pressure measurement.

Ambulatory blood pressure measurement or monitoring (ABPM) is another type of out-of-office BP measurement, which provides intermittent BP records throughout the day when the subjects stay in their usual environment. The ambulatory BP device is fitted on an individual’s non-dominant arm and then programmed to work spontaneously at 20–30 min intervals (Figure 2). Like HBPM, this technique can avoid white-coat effects from office BP measurements and can detect masked hypertension. The advantage of ABPM over HBPM is the obtaining of nighttime BP and the rising patterns of BP around the awakening period, the so-called morning BP surge, and provides BP records throughout the whole day, so BP variability can be detected and clarified.

The difference between daytime and nighttime BP can be categorized as shown in Table 3. Individuals with reduced-dippers and sometimes risers have higher cardiovascular (CV) risk as compared with those who are dippers [9, 10]. The absolute value of nighttime BP is also an important consideration. Nocturnal hypertension, as defined by average nighttime BP at least 120/70 mmHg, is associated with increased CV risk. Nighttime BP has better correlation with CV events than daytime or office BP [10]. It has been demonstrated that 74.0% of Thai hypertensive subjects treated at a university hospital have reduced dipping or rising BP patterns [11]. ABPM also helps to confirm hypotension due to excessive BP lowering and to assess extreme BP changes in patients with autonomic failure. When there is discordance between office and home BP, ABPM should be performed to make a precise diagnosis and assist in delivering the proper treatment. We recommend ABPM to determine specific diurnal BP patterns, nocturnal hypertension, morning BP surge, and BP variability (*Strength of Recommendation I, Quality of Evidence A*).

**Table 3.** Diurnal BP patterns as identified by ABPM and related CV risk

BP patterns	Nighttime BP lowering as compared with daytime BP (%)	CV risk
Normal dipping	>10–20	Notincreased
Extreme dipping	>20	Debatable risk
Reduced dipping	1–10	Increased risk
Rising	Nighttime BP increases	Increased risk

1 ABPM, ambulatory blood pressure measurement; BP, blood pressure; CV, cardiovascular.

THS’ recommendations for BP measurement are summarized in Table 4.

**Table 4.** Recommendations for BP measurement

Recommendations	Strength of recommendations	Quality of evidence
All types of BP measurement, including office, home, and ambulatory BP measurement, are recommended for the diagnosis and treatment of hypertension.	I	A
Only validated BP measuring devices with an appropriate size of upper arm cuff should be used.	I	A
Standardized procedure for BP measurement must be followed to obtain reliable BP records.	I	A
In the first visit, BP should be measured simultaneously in both arms. If an interarm SBP difference >10 mmHg is detected and confirmed with repeated measurements, the arm with the higher BP should be used for subsequent evaluation.	I	A
If interarm SBP difference >15–20 mmHg is detected and confirmed, further investigations to diagnose arterial disease of the upper extremities are recommended.	I	A
Orthostatic hypotension should be screened in subjects with DM, elderly, or with orthostatic symptoms.	IIa	C
BP measurement with the auscultatory technique is preferred in subjects with cardiac arrhythmia, and it is recommended to use the average BP from multiple BP records.	IIa	C
HBPM is advised in all treated hypertensive cases because it will enhance medication adherence, reduce white-coat effect, and detect masked uncontrolled hypertension. Upper arm devices are recommended, but wrist devices may be allowed in markedly obese subjects.	I	A
Cuffless devices are not recommended for clinical use.	III	C
ABPM should be used to determine specific diurnal BP patterns, nocturnal hypertension, morning BP surge, and BP variability.	I	A

1 ABPM, ambulatory blood pressure monitoring; BP, blood pressure; DM, diabetes mellitus; HBPM, home blood pressure measurement; SBP, systolic blood pressure

### Definition of hypertension

According to the previous 2019 THS guidelines [12] and current international guidelines [13–16], hypertension is defined based on repeated office SBP values of at least 140 mmHg and/or DBP of at least 90 mmHg. However, there is a continuous relationship between BP and CV, renal adverse events, and mortality starting from an office SBP >115 mmHg and DBP >75 mmHg [17]. In the Asian population, the CV risks were shown to increase even with BP <140/90 mmHg [18–23]. Furthermore, RCTs have shown that lowering BP to <130/80 mmHg can reduce cardiovascular diseases (CVD) [24, 25]. These THS guidelines change the category of BP between 130–139 mmHg and/or 80–89 mmHg from the term high-normal BP to BP at risk to emphasize the importance of the increasing CV risk in this BP range (*Strength of Recommendation I, Quality of Evidence A*).

### Classification of BP and hypertension severity

The definition and classification of the severity of hypertension, which is determined from the BP measured in clinics, hospitals, or public health centres, is shown in Table 5. The thresholds for diagnosis of hypertension using different methods of BP measurements are summarized in Table 6.

- **White-coat hypertension (isolated office hypertension)** is defined as high office BP (SBP ≥140 mmHg and/or DBP ≥90 mmHg) and normal out-of-office BP.
- **Masked hypertension** is defined as normal office BP (SBP <140 mmHg and DBP <90 mmHg) and high out-of-office BP.

**Table 5.** Definition of BP and classification of the severity of hypertension in adults aged 18 years and older

Category	SBP (mmHg)		DBP (mmHg)
Optimal	<120	and	<80
Normal	120–129	and/or	<80
BP at risk	130–139	and/or	80–89
Grade 1 hypertension	140–159	and/or	90–99
Grade 2 hypertension	160–179	and/or	100–109
Grade 3 hypertension	≥180	and/or	≥110
ISH	≥140	and	<90
IDH	<140	and	≥90

1 BP, blood pressure; DBP, diastolic blood pressure; IDH, Isolated diastolic hypertension; ISH, isolated systolic hypertension; SBP, systolic blood pressure.

**Table 6.** Criteria of hypertension diagnosis in different measurement methods

Measurement method	SBP (mmHg)		DBP (mmHg)
Office BP measurement	≥140	and/or	≥90
HBPM	≥135	and/or	≥85
ABPM			
Average of daytime BP	≥135	and/or	≥85
Average of nighttime BP	≥120	and/or	≥70
Average of 24-h BP	≥130	and/or	≥80

1 ABPM, ambulatory blood pressure measurement; BP, blood pressure; DBP, diastolic blood pressure; HBPM, home blood pressure measurement; SBP, systolic blood pressure.

### Diagnosis of hypertension

Diagnosis of hypertension can be made if high BP, according to the criteria in Table 6, were detected repeatedly, usually by office BP measurement (*Strength of Recommendation I, Quality of Evidence A*). THS recommends the use of standard office BP measurement for better assessment of BP values, as mentioned above (*Strength of Recommendation I, Quality of Evidence A*). The Thai HBPM study showed a significant prevalence of white-coat hypertension and masked hypertension. Therefore, THS encourages the use of out-of-office BP monitoring to achieve a more accurate diagnosis (*Strength of Recommendation I, Quality of Evidence A*). Since high BP detected by out-of-office BP monitoring has better prognostic values than high office BP, if there is a discrepancy between these 2 measurement methods, the diagnosis should be considered based on out-of-office BP (*Strength of Recommendation I, Quality of Evidence A*). If out-of-office BP is not available and the previous BP reading was high, the diagnosis of hypertension should be made if repeated standardized office BP or AOBP in that visit is high (*Strength of Recommendation I, Quality of Evidence A*). Subject with office BP of 130–139/80–89 mmHg and normal out-of-office BP, hypertension should be diagnosed if the subject has hypertension-mediated organ damage (HMOD) (see Section “Evaluation of a patient with hypertension”) or CVD or DM or high CV risk (*Strength of Recommendation I, Quality of Evidence A*).

White-coat hypertension is diagnosed if normal out-of-office BP is detected in subjects with high office BP. In subjects with office BP between 130–139/80–89 mmHg, out-of-office BP monitoring should also be performed. If the out-of-office BP is high, masked hypertension is diagnosed.

**Figure 3.**
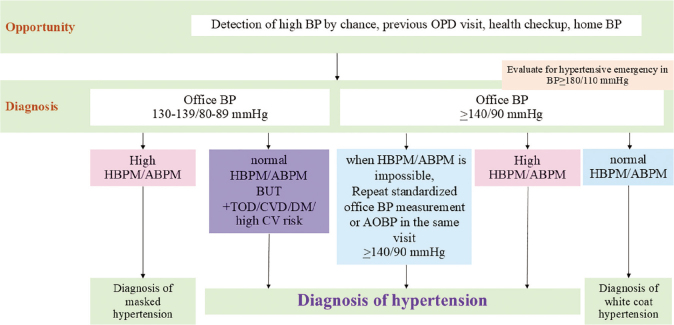
Diagnostic algorithm for hypertension. ABPM, ambulatory blood pressure measurement; AOBP, automated office blood pressure; BP, blood pressure; CV, cardiovascular; CVD, cardiovascular disease; DM, diabetes mellitus; HBPM, home blood pressure measurement; OPD, outpatient department.

If any person has severely elevated BP (grade 3 hypertension) while staying in the hospital, the clinical diagnosis of hypertension should be made even in the inpatient department setting (Strength of Recommendation I, Quality of Evidence A). In subjects with non-severely elevated BP during hospital admission, the follow-up schedule should be made after discharge, to properly screen for possible hypertension (Strength of Recommendation I, Quality of Evidence A). The diagnostic algorithm for hypertension is summarized in Figure 3.

### Evaluation of a patient with hypertension

The purpose of the patient evaluation in hypertension is to identify factors contributing to the development of hypertension (such as diet, physical inactivity, concomitant medications, or family history), to screen for secondary causes of hypertension, to identify concomitant CV risk factors (e.g., DM, dyslipidemia, obesity), to establish whether there are evidences of HMOD or existing CV, or renal disease and, to estimate the patient’s total CV risk to guide threshold for treatment initiation, to guide BP target and the use of statins. Patient evaluation should be initiated starting from individuals with BP at risk and every patient with hypertension [12–16].

### Clinical evaluation

A detailed medical history should include duration of hypertension, record of current and past BP values, antihypertensive medications, including their effectiveness and intolerance, family history of hypertension, history of CVD, kidney disease, and history of erectile dysfunction. History of possible secondary hypertension, concomitant CV risk factors, history, and symptoms of HMOD are also required. Lifestyle evaluation, including exercise levels, body weight changes, diet, smoking status, alcohol consumption, use of licorice, recreational drug use, the individual’s stress level, and sleep history are needed. Thorough physical examination gives important indications of potential causes of secondary hypertension, signs of comorbidities, and HMOD. Physical examination should include body habitus, including waist circumference, fundoscopic examination for hypertensive retinopathy, heart and carotid artery auscultation, palpation of peripheral pulses, neurological examination, and look for signs of secondary hypertension, co-existing diseases, and HMOD. Ankle-brachial index (ABI) and pulse wave velocity (PWV) may be considered if possible (*Strength of Recommendation Ilb, Quality of Evidence B*).

HMOD refers to structural or functional changes in target end organs (heart, blood vessels, brain, eyes, and kidneys) caused by high BP. The recommended laboratory tests are hemoglobin and/or hematocrit, fasting plasma glucose and glycated hemoglobin, blood lipid profiles, serum creatinine for glomerular filtration rate estimation (eGFR), potassium, sodium, uric acid, and urine analysis. Additional investigations of screening for HMOD are recommended in Table 7.

**Table 7.** Recommendations for additional investigations on patients with hypertension

Recommendations	Strength of recommendation	Quality of evidence
**Heart investigations**		
12-lead electrocardiography should be done in every patient with hypertension.	I	B
Transthoracic echocardiography should be done in patients whose ECG is abnormal or in cases with suspected heart disease.	I	B
Transthoracic Echocardiography may be considered in patients suspected of having left ventricular hypertrophy.	IIb	B
**Vascular investigations**		
Carotid artery ultrasound may be considered in patients with carotid bruit, those with cerebrovascular disease, or patients with artery diseases in other parts of the body.	IIb	B
Coronary calcium scan may be considered in patients with intermediate CV risk.	IIb	C
Abdominal aorta ultrasound should be performed in suspected aortic aneurysm.	I	C
PWV may be considered.	IIb	B
ABI may be considered.	IIb	B
**Kidney investigations**		
Serum creatinine and eGFR should be tested in every patient with hypertension.	I	B
Measurement of urine albumin should be done in every patient with hypertension.	I	B
Urine microalbumin should be tested in every patient with hypertension and DM.	I	A
Kidney ultrasound and Doppler can be done in patients with CKD, with albuminuria or suspected of secondary hypertension from renal artery stenosis.	IIa	C
**Eyes investigations**		
Retinal examination should be performed in every patients with very high BP (grade 3 HT) or patients with DM.	I	C
**Brain Investigations**		
CT scan or MRI of the brain can be done for patients with neurological symptoms or cognitive disorders.	IIa	B

1 ABI, ankle-brachial index; BP, blood pressure; CKD, chronic kidney disease; CT, computerized tomographic; CV, cardiovascular; DM, diabetes mellitus; ECG, electrocardiogram; eGFR, glomerular filtration rate estimation; MRI, magnetic resonance imaging; PWV, pulse wave velocity.

#### Total CV risk assessment

THS suggests using the Thai CV risk score for risk evaluation, and the 10-year risk of >10% indicates high CV risk. Risk factor clustering can also be used for total CV risk assessment. Having 3 or more of the following risk factors indicates high CV risk: male sex, age >55 years, cigarette smoking, left ventricular hypertrophy, family history of CVD, albuminuria, DM, other atherosclerotic cardiovascular disease (ASCVD), and total cholesterol/high density lipoprotein ratio >6.

## Prevention and control of hypertension through lifestyle modification

Improving lifestyle behaviors is also fundamental in managing hypertension, regardless of whether patients require pharmacological treatment or not. For hypertensive patients on medication, lifestyle changes can enhance the efficacy of BP-lowering drugs and may even lead to a reduction in medication dosage. Physicians or healthcare professionals should provide guidance on lifestyle modifications to individuals at risk of or already diagnosed with hypertension (*Strength of Recommendation I, Quality of Evidence A*).

### Weight reduction in overweight or obese individuals

For Thai individuals, a normal body mass index (BMI) ranges from 18.5 kg/m^2^ to 22.9 kg/m^2^ and a waist circumference <90 cm (36 inches) for men and <80 cm (32 inches) for women or not exceeding half of their height (a waist-to- height ratio <0.5) for both sexes. To achieve long-term weight reduction and control, it is recommended to focus on cognitive-behavioral therapy processes [26]. In cases where weight loss cannot be achieved through lifestyle modifications alone, consideration may be given to weight loss medications and/or bariatric surgery as indicated.

### Modification of dietary patterns for health

The prototype dietary pattern for reducing BP is the Dietary Approaches to Stop Hypertension (DASH) diet [27, 28]. Moreover, other healthy dietary patterns also have evidence that they can contribute to reducing BP levels, such as a lactoovo vegetarian diet (which incorporates eggs and dairy products as protein sources), the Mediterranean diet [29]. These diets emphasize the consumption of unprocessed foods, whole grains, unrefined carbohydrates, legumes, vegetables, and fruits. They also focus on low-fat protein sources such as lean meats, plant-based proteins, low-fat dairy, and dairy products. However, it is not recommended to rely on potassium and/or magnesium supplements in the form of dietary supplements to reduce BP. For chronic kidney disease (CKD) patients, it is advisable to seek guidance on appropriate dietary recommendations from a physician or dietitian. It is also advisable to avoid dietary supplements or herbal extracts that may potentially increase BP, such as Ma Huang (Ephedra), licorice, bitter orange, and Yohimbe.

### Limiting salt and sodium intake in the diet

Following the recommendations of the World Health Organization (WHO), it is advised to consume no >2 g of sodium per day to prevent non-communicable diseases (NCDs) and help lower BP in individuals with hypertension. Increasingly strict sodium limitations to no >1.5 g/d may further aid in reducing BP. A sodium intake of 2 g is equivalent to approximately 1 teaspoon of salt (5 g of sodium chloride) or 3 teaspoons of fish sauce or soy sauce. Using salt substitutes can be an alternative option for reducing sodium intake. These products often partially replace sodium salts with potassium salts; therefore, patients with CKD should receive appropriate advice from a physician or dietitian. It is also recommended to consume fresh, minimally processed foods and avoid highly processed foods and excessive seasoning to reduce or prevent excessive sodium intake.

### Increasing physical activity and regular exercise

Regular aerobic exercise with appropriate intensity and duration is recommended, with no >2 consecutive days of rest. Individuals can choose from various intensity levels of exercise, including:

- **Moderate intensity:** Exercise that elevates heart rate to 50.0%–70.0% of maximum heart rate (calculated as 220 minus age). This level of exercise should be maintained for an average of 150 min/week.
- **Vigorous intensity:** Exercise that elevates heart rate beyond 70.0% of maximum heart rate. This level of exercise should also be maintained for an average of 75 min/week.

Isometric exercises, such as weightlifting, which involve muscle contraction against resistance, may increase BP levels. Therefore, if BP is not well controlled, it is advisable to consult a physician before starting such exercises. Additionally, it is recommended to reduce daily sedentary time and increase movement whenever possible [26, 30].

### Limiting or abstaining from alcohol consumption

For overall health, individuals who do not consume alcohol should not be encouraged to start, and those who do consume alcohol should limit their intake. Specifically, women should not exceed 1 standard drink per day, and men should not exceed 2 standard drinks per day (1 standard drink is equivalent to a beverage containing approximately 10 g of alcohol). Additionally, it is recommended to have alcohol-free days each week [31].

### Smoking cessation

For individuals who smoke, it is recommended to quit smoking, including both traditional cigarettes, e-cigarettes (vaping), and waterpipe smoking [32, 33]. Physicians or healthcare professionals should advise patients to quit smoking or encourage them to feel motivated to quit smoking (*Strength of Recommendation I, Quality of Evidence A*). For individuals who do not smoke, it is advisable to avoid second-hand smoke exposure.

### Emotional stress management

Emotional stress increases the risk of hypertension and CVD [34, 35]. It is recommended to manage stress appropriately, along with using complementary techniques such as meditation, breathing exercises, music, and yoga [36–39].

### Avoidance of environmental factors that may increase BP

Examples include avoiding places with noise pollution and air pollution [40, 41] *(Strength of Recommendation IIa, Quality of evidence C*).

Recommendations for lifestyle modifications to control and prevent hypertension are summarized in Table 8.

**Table 8.** Recommendations for lifestyle modifications to control and prevent hypertension

Recommendations	Strength of recommendations	Quality of evidence
Weight reduction in over-weight or obese individuals	I	A
Adopting a healthy eating pattern as a routine	I	A
Dietary sodium restriction	I	A
Regularly increasing physical activity and/or engaging in aerobic exercise	I	A
Smoking cessation and avoiding passive smoking	I	A
Limiting the quantity of alcoholic beverages	I	A
Avoid noise pollution and air pollution	IIa	C
Stress management	IIa	C

## Pharmacological treatment

### Initiation of pharmacological treatment

The decision to start antihypertensive medication(s) in patients with hypertension is based on 4 key consideration factors: the average standardized office BP measurement together with out-of-office BP measurement if available, the individual’s CV risk level, co-existing disease(s) in that individual, especially CVD and DM, and lastly the status of HMOD. BP threshold and use of risk estimation to guide pharmacological treatment of hypertension are summarized in Table 9.

**Table 9.** BP treatment threshold and use of risk estimation to guide pharmacological treatment of hypertension

Recommendations	Strength of recommendations	Quality of evidence
Use of antihypertensive medication(s) is recommended in patients with clinical CVD, patients with DM, individuals with an estimated 10-year	I	A for SBP
CVD risk of 10% or higher, and an average office SBP of 130 mmHg or higher and/or an average office DBP of 80 mmHg or higher		C for DBP
Use of antihypertensive medication(s) is recommended in adults with office SBP of 140 mmHg or higher and/or office DBP of 90 mmHg or higher	I	C

1 BP, blood pressure; CVD, cardiovascular disease; DBP, diastolic blood pressure; DM, diabetes mellitus; SBP, systolic blood pressure.

### Target BP level

Suggested target BP levels for drug treatment are summarized in Tables 10 and 11. Nevertheless, there are 2 points of caution. First, the patients’ BP should initially be lowered to under 140/90 mmHg, and if they show good tolerance, then BP should be further reduced to 130/80 mmHg or less. Second, data show some detrimental effects of BP that is too low, especially for elderly, frail patients, patients with pre-existing CVD or comorbidity. For some of these patients, the treatment target should be individualized.

**Table 10.** Office BP targets for patients with hypertension

Recommendations	Strength of recommendations	Quality of evidence
**Patients 18–64 years old**		
Office BP should be lower to <130/80 mmHg.	I	A
**Patients 65–79 years old**		
Office BP should be initially lower to <140/90 mmHg.	I	A
Further office BP lowering to <130/80 mmHg should be considered if treatment is well tolerated.	I	B
**Patients 65–79 years old with ISH**		
Office SBP should be initially lowered in the 140–150 mmHg range.	I	A
Further reduction of office SBP in the 130–139 mmHg range may be considered if well tolerated, albeit cautiously if office DBP is already below 70 mmHg.	IIb	B
**Patients ≥80 years old**		
Office SBP should be lowered in the 140-150 mmHg range and DBP to <80 mmHg.	I	A
Further reduction of office SBP in the 130-139 mmHg range may be considered if well tolerated, albeit cautiously if office DBP is already below 70 mmHg.	IIb	B
**General Recommendations**		
In frail patients, the treatment office BP target should be individualized.	I	C
Do not aim office SBP target below 120 mmHg or an office DBP target below 70 mmHg.	III	C

1 BP, blood pressure; DBP, diastolic blood pressure; ISH, isolated systolic hypertension; SBP, systolic blood pressure.

**Table 11.** Home BP targets for patients with hypertension

Recommendations	Strength of recommendations	Quality of evidence
Average home BP should be lower to <130/80 mmHg.	I	B
Further home SBP lowering to <125/75 mmHg can be considered in patients 18–65 years old, patients with DM, with CVD or with high CV risk.	IIa	B
Average home BP of patients 65–79 years old and patients with history of stroke should be <135/85 mmHg.	I	C
Average home BP below 140/80 mmHg is acceptable for patients 80 years or older.	IIb	C

1 BP, blood pressure; CV, cardiovascular; CVD, cardiovascular disease; DM, diabetes mellitus; SBP, systolic blood pressure.

### Selection of antihypertensive medication

Five major drug classes are recommended as first-line agents for the treatment of hypertension. These 5 major drug classes are angiotensin converting enzyme inhibitors (ACEis), angiotensin receptor blockers (ARBs), beta-blockers, calcium-channel blockers (CCBs), and thiazide/thiazide-like diuretics. The selection of 5 major drug classes as first-line agents was based on the following criteria: 1. a proven ability to reduce BP as monotherapy, 2. evidence from RCTs that they can reduce morbidity and mortality from high BP, and 3. a favorable safety and tolerability profile. To select the appropriate BP medication, physicians have to consider the pre-existing comorbidities other than high BP, as well as the contraindications of each class of drugs.

In the year 2021, angiotensin receptor-neprilysin inhibitor (ARNI) was approved for the treatment of hypertension in Thailand. ARNI is a combination of an ARB (valsartan) and a neprilysin inhibitor (sacubitril), which simultaneously blocks the effects of angiotensin II at the AT-1 receptor and inhibits the degradation of natriuretic peptides, thus promoting peripheral vasodilatation and reducing BP [42]. In a recent meta-analysis of 10 studies including 5,931 hypertensive patients, ARNI reduced office SBP by 6.5 mmHg and DBP by 3.3 mmHg compared with placebo [43]. 24-h SBP and DBP were also reduced by 7.0 and 3.2 mmHg, respectively. Because of a lack of evidence to demonstrate its efficacy to reduce overall CV events in patients with hypertension, it cannot be recommended as 1 of the first-line agents for hypertension therapy. Nevertheless, ARNI may be an appropriate choice, however, in patients with true resistant hypertension (*Strength of Recommendations Ilb, Quality of Evidence B*) and should be used in patients with heart failure with reduced ejection fraction (HFrEF) instead of a renin angiotensin system (RAS) blocker. (*Strength of Recommendations I, Quality of Evidence A*).

Selective sodium-glucose cotransporter-2 inhibitors (SGLT2i) have demonstrated significant BP-lowering effects through various mechanisms such as natriuresis and reduction of sympathetic tone [44]. A meta-analysis of 43 RCTs with 22,528 diabetic patients confirmed the efficacy of SGLT2i in BP lowering [45]. Glucagon-like peptide 1 receptor agonists (GLP1-RA) also have potential for BP reduction. The reduction in BP, shown in randomized clinical trials, was observed early in the treatment, before any significant weight loss occurred, suggesting independent action of GLP1-RA on BP [46]. The proposed mechanisms for its effects might include natriuresis, direct vasodilatation, decreased sympathetic activity, or improved endothelial function [47]. However, there are no RCTs of these medications dedicated to general hypertensive patients, and they have not been approved for BP reduction indication in Thailand. However, they can be used as an ancillary treatment in hypertensive patients with comorbidities that indicate the use of SGLT2i or GLP1-RA. (*Strength of Recommendation IIa, Quality of Evidence A*).

### Antihypertensive drug combinations

We recommend the use of 2 antihypertension drugs as a single-pill combination (SPC) in hypertensive patients wherever and whenever available (*Strength of Recommendation I, Quality of Evidence A*), because reducing the number of pills to be taken daily can improve adherence to treatment and increase the rate of BP control. Recently, the START-Study has shown that antihypertensive combination therapy using SPC could reduce all-cause mortality and CV events when compared to identical drugs prescribed as multi-pill [48]. The THS recommendations for antihypertensive drug treatment are summarized in Table 12.

**Table 12.** Recommendations for antihypertensive drug treatment

Recommendations	Strength of recommendations	Quality of evidence
Medications to start treatment of hypertension should be selected from 5 major drug classes, which include ACEis, ARBs, beta-blockers, CCBs, and diuretics (thiazides and thiazide-like diuretics). These drugs and their combinations are recommended as the basis of antihypertensive treatment.	I	A
A two-drug combination should be started for most patients. Preferred combinations are RAS blockers (ACEI or ARB) with a CCB or thiazide/thiazide-like diuretic. Other combinations of the 5 major drug classes can also be used as appropriate.	I	A
Initiation with 1 drug should be considered in patients with: Advance age, General weakness and/or frailty, Low starting BP (140–149/90–95 mmHg) with low CV risk, BP at risk and very high CV risk.	I	C
If BP cannot be controlled with the initial 2-drug combination using the best tolerated dose of the respective components, treatment should be increased to a 3-drug combination, usually a RAS blocker + CCB + thiazide/thiazide-like diuretic. However, 1 of the 3 components should be a diuretic and preferably a thiazide-like diuretic.	I	A
If BP cannot be controlled with a 3-drug combination by using the maximum recommended and/or tolerated dose of the respective components, which 1 component should be a diuretic, it is recommended to treat the patient as resistant hypertension.	I	A
The use of medication that is a combination of 2–3 different drug classes in a single pill should be preferred at any treatment step.	I	A
The combination of 2 RAS blockers is not recommended, due to increased risk of adverse events, especially AKI, hyperkalemia.	III	A
ARNI may be an appropriate choice, instead of a RAS blocker, in patients with resistant hypertension.	IIb	B
Selective SGLT2is or GLP1-RAs can be used as an ancillary treatment in hypertensive patients with comorbidities that indicate their use.	IIa	A
SGLT2is and/or GLP1-RAs are not recommended solely for the purpose of BP reduction	III	C

1 ACEis, angiotensin converting enzyme inhibitors; AKI, acute kidney injury; ARB, angiotensin receptor blockers; ARNI, angiotensin receptor-neprilysin inhibitor; BP, blood pressure; CCBs, calcium-channel blockers; CV, cardiovascular; GLP1-RA, glucagon-like peptide 1 receptor agonists; RAS, renin angiotensin system; SGLT2i, sodium-glucose cotransporter-2 inhibitors.

### Prescribing of antihypertensive drugs

The primary goal of antihypertensive treatment is to provide BP control over 24 h. Antihypertensive drugs that have a long duration of action that can cover the 24 h with a single daily administration should be preferred. Most of the evidence on the benefit of BP-lowering treatment has come from clinical trials using morning dosing of antihypertensive agents. The Treatment in Morning versus Evening (TIME) study, published in 2022, showed no significant difference in the primary outcome (hospitalization for major CV events and vascular death) between groups of patients randomized to the evening-dosing and the morning-dosing [49]. The reported non-adherence to therapy was significantly higher with evening versus morning dosing (39.0 versus 22.5%, *P* < 0.0001), with no reported safety concerns. THS suggests the appropriate timing of prescribed antihypertensive drugs in Table 13.

**Table 13.** Suggestions for appropriate timing of antihypertensive drug administration

Recommendations	Strength of recommendations	Quality of evidence
Patients can choose as to when to take their antihypertensive medication, in the morning or before bedtime.	I	C
Physicians may advise bedtime dosing in patients with documented high nocturnal BP	IIb	C
In general, THS recommend taking antihypertensive medication in the morning, as adherence to medication is better than at nighttime.	I	C

1 BP, blood pressure; THS, Thai Hypertension Society.

### Management of white-coat hypertension

Individuals with white-coat hypertension have less prevalence of HMOD than those with sustained hypertension and have a lower risk of CV events. However, compared with true normotensive individuals, persons with white-coat hypertension have increased adrenergic activity [50], a greater prevalence of metabolic risk factors, a more frequent asymptomatic HMOD [51], a higher risk of new-onset DM, and are more likely to progress to sustained hypertension and have a higher risk of CV mortality [52–54]. Ideally, out-of-office BP measurements should include both ABPM and HBPM because the 2 measurement values can give discrepant results. Although the CV risk of people with white-coat hypertension, in whom both ABPM and HBPM are normal, appears to be low and close to that of normotensive individuals [55], they need to regularly follow up. Usually, antihypertensive drug treatment effectively lowers office BP in white-coat hypertensive patients, but the effect on out-of-office BP is small and variable [56]. Whether patients with white-coat hypertension should receive antihypertensive drugs is still unresolved; however, drug treatment can be given in such patients with HMOD and/or high CV risk (*Strength of recommendation IIa, Quality of Evidence C*) as in Table 14.

**Table 14.** Recommendations for management of white-coat hypertension

Recommendations	Strength of recommendations	Quality of evidence
Diagnosis of white-coat hypertension should be made and confirmed by out-of-office BP measurements, particularly in individuals with office BP in grade 1 hypertension.	I	B
Assessment of CV risk factors and HMOD is recommended in individuals with white-coat hypertension.	I	B
Patients with white-coat hypertension should be advised to change their improper lifestyle and reduce CV risk.	I	B
Patients with white-coat hypertension should be follow-up to screen for new HMOD.	I	B
Out-of-office BP measurements should be repeated from time to time, during follow-up, to timely identify sustained hypertension.	I	B
Patients with white-coat-hypertension with HMOD and/or high CV risks can be considered for antihypertensive drug therapy.	IIa	C

1 BP, blood pressure; CV, cardiovascular; HMOD, hypertension-mediated organ damage.

### Management of masked hypertension

The optimal approach for detecting masked hypertension is challenging. An office BP in the BP at risk range is associated with a higher likelihood of masked hypertension. Masked hypertension has been reported to be associated with HMOD and has an increased risk of developing DM and sustained hypertension [53, 57]. Meta-analysis has shown that the CV risk in patients with masked hypertension is substantially greater than that of individuals with true normotension and even close to the risk of patients with sustained hypertension [58, 59]. No therapeutic RCT has ever been done on masked hypertension. However, given the adverse prognostic nature of masked hypertension, we recommend the management of masked hypertension as in Table 15.

**Table 15.** Recommendations for management of masked hypertension

Recommendations	Strength of recommendations	Quality of evidence
Individuals with BP at risk should have out-of-office BP measurement by ABPM and/or HBPM, to identify masked hypertension	I	B
In patients with confirmed masked hypertension, stringent lifestyle interventions and close follow-up to timely identify sustained hypertension, and to detect new HMOD are recommended.	I	C
Antihypertensive drug therapy for individuals with confirmed masked hypertension can be considered if CV risk is high and/or having HMOD.	IIa	C

1 ABPM, ambulatory blood pressure measurement; BP, blood pressure; CV, cardiovascular; HBPM, home blood pressure measurement; HMOD, hypertension-mediated organ damage.

## Hypertension in patients with comorbidities

### Treatment of hypertension in patients with stable coronary artery disease

Increased BP, starting from 115/75 mmHg, is associated with coronary artery disease (CAD)-related mortality [17]. BP reduction can decrease the incidence of CAD in hypertensive individuals regardless of the type of antihypertensive medication used [60]. Many randomized placebo-controlled trials involving patients with CAD showed that strict SBP lowering prevented complications of CAD. There were concerns about the possibility that an excessive lowering of DBP may increase the incidence of CV events in patients with CAD, the so-called J-curve phenomenon. However, from a systematic review and meta-analysis of 7 randomized placebo-controlled trials enrolling 34,814 CAD patients who achieved DBP <80 mmHg included in the drug-intervention group, the intervention was associated with an 11% reduction in coronary revascularization and a 31.0% reduction in heart failure (HF) [61]. Also, a meta-analysis of 4 RCTs, in which the achieved DBP was <75 mmHg, showed that the drug intervention was associated with a 22.0% reduction in HF [61] (Table 16).

**Table 16.** Recommendations for patients with stable CAD

Recommendations	Strength of recommendations	Quality of evidence
In patients with stable CAD, antihypertensive drug treatment should be initiated in the office, with BP at risk range of 130–139 mmHg and/or 85–89 mmHg.	I	A
In patients with stable CAD, the treatment targets should be the same as in the general hypertensive population.	I	A
In patients with stable CAD and low office DBP (<70 mmHg), if office SBP is still well above the target values, the office SBP can be cautiously lowered.	IIa	C
In patients with CAD and low office DBP, office SBP lowering can be considered while monitoring tolerability, such as symptoms and signs of organ ischemia, especially in elderly patients.	IIa	C
ACEi and/or beta-blocker is recommended in patients with stable CAD and high BP. However, ARB can replace ACEi if not tolerated.	I	A
In symptomatic CAD patients with angina, beta-blockers, both DHP-CCB and non-DHP CCB, are recommended for the treatment of hypertension.	I	A
Lowering heart rate of CAD patients with hypertension to between 60 bpm and 80 bpm can be useful, for which a beta-blocker or a non-DHP-CCB should be prescribed	I	B

1 ACEis, angiotensin converting enzyme inhibitors; ARB, angiotensin receptor blockers; BP, blood pressure; CAD, coronary artery disease; DBP, diastolic blood pressure; DHP-CCB, dihydropyridine calcium channel blockers; SBP, systolic blood pressure.

### Treatment of hypertension in patients with HF

HF is a significant health problem in Thailand. Between 2008 and 2013, the HF hospitalization rate in Thailand gradually increased from 61,594/year to 80,246/year [62]. Data from an HF registry and a real-world hospital database in Thailand consistently show that hypertension is a significant comorbidity in HF patients. According to the Thai Acute Decompensated Heart Failure Registry (ADHERE), which collected data from 1,612 patients with acute decompensated heart failure (ADHF) admitted during 2006–2007, the prevalence of hypertension in Thai HF patients was around 65.0% [63]. Ten-year follow-up data from the ADHERE registry shows that the mortality rate was above 70%; having hypertension at baseline was more common in those who died compared to those who survived [64].

#### Prevention of HF in patients with hypertension

High BP increases left ventricular afterload, activates renin angiotensin aldosterone and the sympathetic nervous system, causing hypertrophy, damage, and fibrosis of cardiac myocytes. High BP can also impair coronary reserve and aggravate myocardial infarction, resulting in ventricular remodeling, cardiac dysfunction, and HF [65]. A recent publication from the BP Lowering Trialists’ collaboration that analyzed 344,716 individual patient data from 48 randomized control trials shows that a reduction of SBP by 5 mmHg can reduce the risk of HF by 13% irrespective of previous diagnosis of CVD [66] (Table 17).

**Table 17.** Recommendations for HF prevention in hypertension

Recommendations	Strength of recommendations	Quality of evidence
Treatment of hypertension is recommended to effectively prevent HF	I	A
All major antihypertensive drug classes can be used in hypertension treatment for the prevention of HF	I	A

1 Hr, heart failure.

The reduction of HF events is even more pronounced in 2 RCTs comparing intensive and standard BP lowering. In the Systolic Blood Pressure Intervention Trial (SPRINT), a 36.0% lower rate of ADHF events was found in the group with a SBP target of <120 mmHg compared to the group with a SBP target of <140 mmHg. This beneficial effect was consistent across all the key prespecified subgroups [67]. In the Strategy of Blood Pressure Intervention in the Elderly Hypertensive Patients (STEP) study, the SBP target of 110–129 mmHg (intensive treatment) was compared to a target of 130–150 mmHg (standard treatment) in Chinese elderly, and ADHF was reduced by 73.0% in this intensively treated population [68].

### Pharmacological treatment of hypertension in HFrEF

According to contemporary randomized control trials in HF, 4 drug classes are recommended for treatment of HFrEF: ACEi or ARB or ARNI, beta-blocker, spironolactone, and SGLT2i [69]. ACEi, ARB, or ARNI and beta-blockers are part of the basic antihypertensive treatment strategy in Thailand. Spironolactone is recommended in patients with resistant hypertension. Curiously, SGLT2i has shown an important additional aspect that they can reduce office BP and average BP from ABPM significantly in randomized controlled CV outcome trials in patients with type 2 DM [70]. Apart from these 4 major drug classes, diuretics are recommended to manage fluid balance and reduce congestion in HF patients. Thiazide or thiazide-like diuretics are preferable if fluid retention is not a significant problem. Loop diuretic, on the other hand, is given to patients who suffer from fluid retention, especially if they have pulmonary edema or advanced CKD (eGFR <30 mL/min/1.73 m^2^) (Table 18).

**Table 18.** Recommendations for pharmacological treatment of hypertension in HFrEF

Recommendations	Strength of recommendations	Quality of evidence
In patients with HFrEF, it is recommended to combine drugs with proven HF outcome benefits, including ACEI or ARB or ARNI, beta-blocker, mineralocorticoid receptor antagonist, and SGLT2i, if not contraindicated and well tolerated	I	A
If patients remain with uncontrolled hypertension from the 4 major drug classes and a diuretic, a DHP-CCB should be added for BP control	I	B
Use of non-DHP-CCB is not recommended in HFrEF	III	C

1 ACEI, angiotensin converting enzyme inhibitors; ARB, angiotensin receptor blockers; ARNI, angiotensin receptor-neprilysin inhibitor; DHP-CCB, dihydropyridine calcium channel blockers; HF, heart failure; HFrEF, heart failure with reduced ejection fraction; SGLT2i, sodium-glucose cotransporter-2 inhibitors.

#### Pharmacological treatment of hypertension in heart failure with preserved ejection fraction

Heart failure with preserved ejection fraction (HFpEF) is more common than HFrEF but usually overlooked. Data from the Thai ADHERE registry show that the majority of ADHF patients had HFpEF (61.5%), but the mortality rate of HFpEF was not different from HFrEF [64]. Hypertension causes left ventricular hypertrophy and diastolic dysfunction, making it the most frequent precursor and comorbidity for HFpEF. Treatment of hypertension leads to LV mass reduction, afterload reduction, and improvement of diastolic function. In a sub-study of the Prospective Comparison of ARNI with ARB Global Outcomes in HF with Preserved Ejection Fraction (PARAGON-HF) trial, apparent resistant hypertension can be found in 15.0% of the study population. ARNI has a more potent effect in HFpEF patients with resistant or refractory hypertension compared to valsartan [71]. SGLT2i has been shown to improve outcomes in dedicated RCTs on HFpEF in both non-diabetic and diabetic patients and could be used to treat this condition [72]. Spironolactone has the potential to reduce outcomes in the Treatment of Preserved Cardiac Function Heart Failure with an Aldosterone Antagonist (TOPCAT) study. It can be considered as an adjunct anti-hypertensive therapy in patients with HFpEF, regardless of diagnosed resistant hypertension, particularly in patients with left ventricular ejection fraction on the lower end of the spectrum [73]. These medications are also recommended as pharmacological treatment for patients with HFpEF in the 2023 Thai HF guideline [74] (Table 19).

**Table 19.** Recommendations for pharmacological treatment of hypertension in HFpEF

Recommendations	Strength of recommendations	Quality of evidence
Treatment of hypertension is recommended in patients with HFpEF	I	A
Substitution of a RAS-blocker by an ARNI can be considered, particularly in those with apparent resistant hypertension	IIb	B
Treatment with spironolactone can be considered regardless of diagnosed resistant hypertension, particularly in patients with low LVEF on the lower end of the spectrum	IIb	B

1 ARNI, angiotensin receptor-neprilysin inhibitor; HFpEF, heart failure with preserved ejection fraction; LVEF, left ventricular ejection fraction; RAS, renin angiotensin system.

#### BP target in hypertensive patients with HF

Given the stronger association between hypertension and CVDs in Asians compared to Caucasians, measures to control BP, to prevent and slow the progression to later stages of HF, are a priority for Asian populations [75]. Hypertensive patients with HFrEF and HFpEF should also aim for a similar target BP of <130/80 mmHg [76] (*Strength of Recommendation I, Quality of Evidence C*). In patients with advanced or refractory HF (stage D HF), physicians should maximize BP-lowering medications by using guideline-directed medical therapy (GDMT) before adding other drug classes, provided they are not contraindicated and are well tolerated (*Strength of Recommendation I, Quality of Evidence C*). This approach could potentially improve prognosis. However, the target BP in stage D HF depends on the patient’s tolerability and expected longevity [77] (Table 20).

**Table 20.** Recommendations for target BP in hypertensive patients with HF

Recommendations	Strength of recommendations	Quality of evidence
In hypertensive patients at risk for HF, the recommended target BP is <130/80 mmHg	I	A
In patients with HFrEF and hypertension, the recommended target SBP is <130 mmHg	I	C
In patients with HFpEF and hypertension, the recommended target SBP is <130 mmHg	I	C
In stage D HF, GDMT should be maximized to improve hemodynamics, if not contraindicated and well tolerated	I	C

1 BP, blood pressure; GDMT, guideline-directed medical therapy; HF, heart failure; HFrEF, heart failure with reduced ejection fraction; HFpEF, heart failure with preserved ejection fraction; SBP, systolic blood pressure.

### Hypertension in patients with CKD, renovascular disease, and kidney transplantation

#### Hypertension in patients with CKD

CKD is diagnosed in anyone with eGFR <60 mL/min/1.73 m^2^ or kidney structure abnormalities, such as albuminuria >30 mg/g creatinine persisting for >3 months. Hypertension is a strong independent risk factor for developing CKD and for the progression of CKD to end-stage kidney disease (ESKD) [78]. In both diabetic and non-diabetic CKD with hypertension, BP-lowering treatment slows the decline of kidney function and reduces the risk of ESKD and CV outcomes. A sub-analysis of the systolic blood pressure intervention trial in chronic kidney disease (SPRINT CKD) subpopulation showed a lower total mortality rate among patients in the intensive BP treatment group (target SBP <120 mmHg) [79]. However, rates of serious adverse events, including hypotension, syncope, electrolyte abnormalities, and acute kidney injury (AKI), were higher in the intensive-treatment group compared to the standard-treatment group. A meta-analysis of studies in CKD patients found that more intensive versus less intensive BP control resulted in a 14.0% reduction in the risk of all-cause mortality [80]. An analysis of the combined trials from the Modification of Diet in Renal Disease Study (MDRD) and the African American Study of Kidney Disease and Hypertension Study (AASK) indicated that a lower target BP was associated with significant reductions in the risks of ESKD and mortality in the CKD population, especially among those with proteinuria of ≥0.44 g/g creatinine [81]. For CKD patients, particularly those with albuminuria and high CV risk, the recommended office target BP could be 120–130/70–79 mmHg (*Strength of Recommendation IIa, Quality of Evidence A*) (Table 21).

**Table 21.** Recommendations for patients with CKD, renovascular disease, and kidney transplantation

Recommendations	Strength of recommendations	Quality of evidence
BP should be monitored at all stages of CKD, because hypertension is the most important risk factor for ESKD.	I	A
Immediate lifestyle interventions and antihypertensive drug treatment should be done in most patients with CKD, regardless of the CKD stage, if BP is ≥140/90 mmHg.	I	A
For CKD patients, especially those with albuminuria and high CV risk, the office target BP could be 120–130/70–79 mmHg.	IIa	A
CKD patients with an albumin-to-creatinine ratio ≥30 mg/g creatinine should be prescribed an ACEi or an ARB as the first-line medication.	I	A
CKD patients with albuminuria <30 mg/g creatinine should be prescribed all major antihypertensive drug classes, including an ACEi, an ARB, a beta-blocker, a CCB, and a thiazide/thiazide-like diuretic.	I	B
The combination of ACEi and ARB should not be prescribed for BP control.	III	B
The same office BP targets as in the hypertensive CKD population apply also to patients with renovascular disease.	IIa	B
ACEis or ARBs may be considered for the treatment of hypertension associated with renovascular disease if well-tolerated and under close monitoring.	IIb	B
The office BP may be lowered to <130/80 mmHg in kidney transplant patients.	IIb	B
ACEi/ARB or DHP-CCB should be used as the first-line antihypertensive agent in adult kidney transplant patients.	I	A

1 ACEis, angiotensin converting enzyme inhibitors; ARB, angiotensin receptor blockers; BP, blood pressure; CCB, calcium channel blockers; CKD, chronic kidney disease; CV, cardiovascular; DHP-CCB, dihydropyridine calcium channel blockers; ESKD, end-stage kidney disease.

Clinical trials in CKD patients, both with and without diabetes, have established that an ACEi or an ARB is the initial treatment of choice for hypertensive CKD patients with significant albuminuria [82–84] because they reduce albuminuria, slow the rate of GFR decline, and lower the risk of doubling serum creatinine or progressing to ESKD. However, ACEi therapy offers no benefits for CKD patients with proteinuria <500 mg/d, even when they are at relatively high risk for progression [85]. An ACEi or an ARB, titrated to the maximum tolerated dose, is recommended for CKD patients with albuminuria. Continue ACEi or ARB therapy unless serum creatinine rises by >30.0% within 4 weeks following the initiation of treatment or an increase in dose. Consider reducing the dose or discontinuing ACEi or ARB in the setting of either symptomatic hypotension or uncontrolled hyperkalemia despite medical treatment, or to alleviate uremic symptoms in CKD stage 5. In CKD patients with hyperkalemia, a potassium binder can be used to maintain normal serum potassium levels (<5.5 mmol/L) to enable optimal treatment with ACEi or ARB to continue [86].

Achieving the recommended BP targets in CKD usually requires combination therapy, which should include an ACEi or ARB along with a CCB or a thiazide/thiazide-like diuretic if eGFR levels are >45 mL/min/1.73 m^2^. For patients with an eGFR between 30 mL/min/1.73 m2 and 45 mL/min/1.73 m2, thiazide/thiazide-like diuretics can generally be replaced by loop diuretics. Mineralocorticoid receptor antagonists are effective in managing refractory hypertension but may lead to hyperkalemia or a reversible decline in kidney function. Dual combinations of ACEi and ARB should be avoided, as this combination therapy increases the risk of hyperkalemia and AKI [87, 88].

#### Hypertension in patients with renovascular disease

Renovascular hypertension is one of the most common forms of secondary hypertension, affecting up to 30% of patients with secondary hypertension [89]. The current consensus is to consider revascularization for patients with documented secondary hypertension due to atherosclerotic renovascular disease, especially in cases involving acute pulmonary edema, progressive CKD, or documented high-grade stenosis (>75%) [90]. The use of ACEi or ARB is now considered as the primary treatment for hypertension in the context of atherosclerotic renovascular disease, and antihypertensive therapy often involves multiple drugs [91].

#### Hypertension in patients with kidney transplantation

High BP is associated with kidney function decline, HMOD, and reduced graft and patient survival [92, 93]. BP targets for hypertension management in kidney transplant patients are extrapolated from data in CKD populations because there are no specific trials that have tested different BP targets on major clinical endpoints. The evidence review found no benefit of ACEi, ARB, or CCB use on all-cause mortality or CV events. However, in a meta-analysis, it was discovered that the risk of graft loss was reduced by 38.0% with ACEis/ARBs [94]. In kidney-transplanted patients, DHP-CCBs have consistently demonstrated benefits, such as improved graft survival and the minimization of the vasoconstrictive effects of calcineurin inhibitors at preglomerular sites. Another meta-analysis showed that CCBs reduced the risk of graft loss by 42.0% [94].

### BP management in patients with stroke

The significance of hypertension as a risk factor for both ischemic and hemorrhagic stroke is well established. Importantly, by effectively reducing BP, the risk of stroke can be reduced. Aggressive BP control through lifestyle modifications and pharmacological interventions can significantly decrease the risk of stroke occurrence and recurrence in hypertensive individuals. Another challenge lies in the management of BP during the acute phase of stroke. In patients with ischemic stroke, remarkably high BP has been linked to hemorrhagic transformation and brain edema, while in patients with intracerebral hemorrhage, high BP may experience hematoma expansion. Conversely, excessively low BP in patients with acute ischemic stroke may be associated with cerebral hypoperfusion and clinical deterioration. Acknowledging this phenomenon emphasizes the need for careful monitoring during the acute phases of stroke. Timely intervention is crucial to reduce complications associated with stroke [95–109] (Table 22).

**Table 22.** Recommendations for BP management in patients with stroke

Recommendations	Strength of recommendations	Quality of evidence
**BP management in patients with acute ischemic stroke**		
In patients with BP >185/110 mmHg, urgent BP reduction should be initiated before starting intravenous thrombolysis.	I	B
In patients with BP >185/110 mmHg, urgent BP reduction can be initiated before mechanical thrombectomy.	IIa	B
Maintaining BP below 180/105 mmHg in the first 24 h after treatment (thrombolysis or mechanical thrombectomy) is recommended.	I	C
It is not recommended to use short-acting nifedipine due to the risk of causing excessive BP reduction, especially in the setting of acute ischemic stroke.	III	C
**Patients who are not candidates for intravenous thrombolysis or mechanical thrombectomy**		
If BP remains >220/120 mmHg, treatment can be initiated to control SBP to be <220 mmHg and DBP <120 mmHg.	IIa	C
**BP in patients with acute sICH**		
SBP >180 mmHg can be reduced by administering antihypertensive medication intravenously.	IIa	B
In patients with sICH with mild to moderate severity, acute lowering of SBP to the target of 140 mmHg is safe and may be considered.	IIb	B
**BP control after acute phase of stroke**		
Patients previously treated for hypertension before the onset of stroke. In this group, resumption 1 of oral antihypertensive medications is recommended.	I	A
Patients were previously treated for hypertension before the onset of stroke. The resumption of oral antihypertensive medications should be initiated before the patient is discharged from the hospital.	IIa	B
Patients who have not been treated for hypertension before the onset of stroke. Oral antihypertensive medications are recommended when BP is >140/90 mmHg	IIa	B
**BP control for secondary stroke prevention**		
After ischemic stroke, oral antihypertensive medication may be administered when BP exceeds 140/90 mmHg, with a target BP range of 120–130/70–80 mmHg.	IIb	B
Patients with intracranial artery stenosis may experience transient ischemic attacks or ischemic strokes associated with a decrease in BP. Lowering BP in this patient group requires special caution, and the appropriate target BP level should be carefully considered on an individual basis	IIb	C
In patients with sICH, it is reasonable to lower BP to 130/80 mmHg for long-term management to prevent recurrence.	IIa	B
It is important to choose an antihypertensive medication that can effectively reach the target BP, 1 as the extent of BP reduction is more significant than the specific type of medication.	I	A

1 BP, blood pressure; DBP, diastolic blood pressure; SBP, systolic blood pressure; sICH, spontaneous intracerebral hemorrhage.

### Treatment of hypertension in patients with peripheral artery disease

The high BP associated with atherosclerosis is a key pathology for the development of peripheral artery disease (PAD). Regular BP monitoring and control of BP can help prevent the development and progression of PAD and reduce the risk of complications associated with this condition [110]. No RCT has been conducted specifically to explore the optimal BP target for preventing PAD. Nonetheless, evidence suggests that SBP below 120 mmHg or above 160 mmHg is associated with a higher risk of PAD-related adverse outcomes, compared to maintaining an SBP within the 120–129 mmHg range [111]. All major BP-lowering medications are equally recommended [111, 112] (Table 23).

**Table 23.** Recommendations for hypertensive patients with PAD

Recommendations	Strength of recommendations	Quality of evidence
In patients with PAD, maintaining BP within the normal range (SBP 120–129 mm Hg) is part of a general strategy for reducing CV risk and may reduce the risk of PAD-related adverse outcomes.	I	B
All major BP-lowering medications, such as diuretics, CCBs, RAS blockers, and beta-blockers are equally recommended.	I	B
For patients with both PAD and hypertension, lifestyle changes especially smoking cessation, and addressing atherosclerotic risk factors are recommended.	I	C

1 BP, blood pressure; CCB, calcium-channel blockers; CV, cardiovascular; PAD, peripheral artery disease; RAS, renin angiotensin system; SBP, systolic blood pressure.

### Treatment of hypertension in patients with diabetes

Hypertension is common in both type 1 and type 2 diabetes. In type 1 diabetes, hypertension usually develops at least 5–10 years after the diagnosis of diabetes and parallels the development of diabetic kidney disease [113–116]. In type 2 diabetes, hypertension is usually associated with metabolic syndrome and may already be present at the time of diagnosis of diabetes [115]. Overall, the prevalence of hypertension in people with diabetes is 60.0%–70.0% [117, 118]. Coexistence of diabetes and hypertension markedly increases the risk of both micro- and macrovascular complications [116]. Hypertension is therefore considered one of the major treatment goals in people with diabetes, in addition to glucose, lipid, and body weight control [116, 119].

Epidemiological studies demonstrated a positive correlation between BP levels and incident CVD in both individuals with and without diabetes [120–122]. Randomized control trials have shown significant benefits of BP-lowering treatment in individuals with SBP of 130 mmHg or higher and additional CV risk factors [123–125]. However, in individuals with diabetes, clinical trials that demonstrated the benefit of reducing BP to <140/90 mmHg were limited. The Action to Control Cardiovascular Risk in Diabetes, Blood Pressure (ACCORD BP) trial is the largest RCT aiming for different SBP targets in people with type 2 diabetes [126]. The primary composite outcome of the study, which included non-fatal MI, non-fatal stroke, or death from CV causes, was not significantly different between the intensive therapy (targeting SBP below 120 mmHg) and standard therapy (targeting SBP below 140 mmHg) groups. However, the prespecified secondary outcome of stroke was significantly reduced by 41.0% in the intensive treatment group. The SPRINT trial [24], which enrolled non-diabetic individuals with increased CV risk to intensive treatment (SBP target of <120 mmHg) versus standard treatment (SBP target of <140 mmHg) demonstrated that the primary composite outcome of MI, acute coronary syndromes, stroke, HF, or death from CV causes was significantly reduced by 25.0% in the intensive treatment group. Despite some apparent discrepant results between the 2 trials, most experts agree that both the ACCORD and SPRINT trials convey the same message of a greater CV benefit with more aggressive BP lowering [127]. Moreover, a recent network meta-analysis of BP control in type 2 diabetes found that the lowest risk of major CVD was found in patients with achieved SBP levels of 120–124 mmHg [128]. Diagnosis and treatment of hypertension in patients with diabetes [28, 119, 129–143] are summarized in Table 24.

**Table 24.** Recommendations for hypertensive patients with diabetes

Recommendations	Strength of recommendations	Quality of evidence
Individuals with diabetes found to have office BP ≥130/80 mmHg should have BP confirmed on a different visit, to diagnose hypertension.	I	A
Hypertension is defined as an office SBP ≥130 mmHg and/or an office DBP ≥80 mmHg based on an average of at least two measurements on at least two occasions.	I	A
Individuals with office SBP ≥180 mmHg and/or DBP ≥110 mmHg, with repeat measurement, should be diagnosed with hypertension at a single visit.	I	B
Individuals with diabetes and hypertension should have HBPM.	I	A
Office BP targets for individuals with diabetes and hypertension should be ≤130/80 mmHg.	I	B
For individuals with BP 130–139/80–89 mmHg, lifestyle intervention such as body weight reduction in overweight or obese individuals, reducing sodium intake, moderation of alcohol intake, and increased physical activity is indicated.	I	A
Pharmacological intervention is indicated for individuals with office BP ≥140/90 mmHg or those who have persistently elevated office BP ≥130/80 mmHg despite lifestyle intervention for at least 3 months.	I	A
Individuals with confirmed office BP ≥150/90 mmHg should have prompt initiation of 2 drugs or a SPC of 2 antihypertensive drugs.	I	A
All first-line classes of antihypertensive agents should be selected to treat hypertension in diabetes.	I	A
In individuals with diabetes and hypertension, ACEis or ARBs are recommended for patients with albuminuria (UACR ≥30 mg/g creatinine).	I	A
Combination of ACEis and ARBs is not recommended.	III	A
SGLT2is or GLP1-RAs are recommended for diabetes individuals with ASCVD and hypertension.	I	A
SGLT2is are recommended for individuals with HF or CKD (eGFR <60 mL/min/1.73 m^2^ or presence of albuminuria) and hypertension.	I	A
GLP1-RAs can be prescribed for individuals with diabetes and HF or CKD and hypertension.	IIa	A

1 ACEis, angiotensin converting enzyme inhibitors; ARB, angiotensin receptor blockers; ASCVD, atherosclerotic cardiovascular disease; BP, blood pressure; CKD, chronic kidney disease; DBP, diastolic blood pressure; eGFR, glomerular filtration rate estimation; GLP1-RA, glucagon-like peptide 1 receptor agonists; HBPM, home blood pressure measurement; HF, heart failure; SBP, systolic blood pressure; SGLT2i, sodium-glucose cotransporter-2 inhibitors; SPC, single-pill combination.

### Treatment of hypertension in patients with obesity

The WHO reports that, in 2020, nearly 1 billion people, or 14.0% of the global population, had obesity (BMI ≥30 kg/m^2^), and Thailand is not immune to this challenge [144]. Similarly, a 2023 WHO global report on hypertension stated that over 1.3 billion adults, or one-third of the worldwide population, are affected by hypertension [145]. The American Heart Association raises concerns about obesity and hypertension as global public health crises [146, 147]. The association between obesity and hypertension is well documented [148–151]. Obesity is linked to hypertension through several mechanisms [152, 153]. These include hyperleptinemia, increased sympathetic activity, and activation of the renin-angiotensin-aldosterone system. These mechanisms lead to vasoconstriction and shift the pressure-natriuresis curve to the right, resulting in salt and water retention. Vascular and kidney dysfunction caused by low-grade inflammation and lipotoxicity also contribute to hypertension in obese individuals. Additionally, obstructive sleep apnea (OSA) is a significant risk factor for hypertension in obese individuals, as it disrupts sleep patterns and oxygen levels, contributing to increased inflammation and sympathetic activity [154–156]. Effective BP control is crucial for obese individuals with hypertension [157]. An important consideration in managing hypertension in obese individuals is the accurate measurement of BP. Appropriate cuff size is emphasized to ensure correct measurement of BP [158, 159].

While target BP goals are generally the same for individuals with and without obesity, achieving these goals can be more challenging in obese individuals. This often requires a more intensive approach of combining comprehensive lifestyle modifications, especially weight reduction, and titrated medication regimens [153, 160–172]. In individuals who, despite lifestyle modifications and obesity pharmacotherapy, cannot significantly reduce body weight, bariatric or metabolic surgery may be considered. Candidates for this procedure are those with a BMI ≥40 kg/m^2^ or those with a BMI of 35-39.9 kg/m^2^ and having comorbid conditions [173, 174]. Individuals with type 2 diabetes and BMI ≥30 kg/m^2^ are also candidates for metabolic surgery if they are not in good glycemic control while on reasonable medical therapy [175]. Before implementing treatment with bariatric surgery, it is essential that individuals fully understand the potential risks and long-term commitments associated with bariatric surgery. Despite various strategies to reduce body weight, many obeseindividuals may still have high BP and thus require antihypertensive medications [176–181]. A summary of recommendations for the treatment of hypertension in patients with obesity is shown in Table 25.

**Table 25.** Recommendations for the treatment of hypertension in patients with obesity

Recommendations	Strength of recommendations	Quality of evidence
In individuals with obesity, weight reduction is fundamental to reduce BP and improve CV outcomes.	I	A
Associated comorbidities that can elevate BP, e.g. OSA, should be aware and appropriately managed.	I	A
All major classes of antihypertensive agents can be used in individuals with obesity or metabolic syndrome.	I	B
Effective medications that do not worsen obesity or metabolic profiles should be selected.	I	A

1 BP, blood pressure; CV, cardiovascular; OSA, obstructive sleep apnea.

### Treatment of hypertension in patients with OSA

The prevalence of OSA in the Thai population was 4.4%-11.4%, depending on the definition of OSA [182]. The prevalence of hypertension in OSA ranges from 35.0%-80.0% [183] and may be related to the severity of obstruction. The pathogenesis of OSA-associated hypertension may be caused by intermittent hypoxia that leads to systemic inflammation and increased oxidative stress [183]. The characteristics of hypertension in OSA are a non-dipping pattern and nocturnal hypertension, which lead to an increased risk of vascular complications [184]. In patients with OSA, the prevalence of masked hypertension and resistant hypertension is higher than in the general population [185]. THS recommendations for treatment of hypertension in patients with **OSA** are summarized in Table 26.

**Table 26.** Recommendations for hypertensive patients with OSA

Recommendations	Strength of recommendations	Quality of evidence
OSA should be suspected in patients who have daytime sleepiness, loud snoring, choking, or interruptions in breathing while sleeping, especially in obese patients.	I	A
Polysomnography should be performed in patients who are suspected of OSA.	I	A
Primary aldosteronism can be screened in OSA patients.	IIa	B
Mineralocorticoid receptor antagonists, ACEis or ARBs should be used to lower BP in hypertensive patients with OSA.	I	A
CPAP can be considered in OSA patients with hypertension.	IIa	B

1 ACEis, angiotensin converting enzyme inhibitors; ARB, angiotensin receptor blockers; BP, blood pressure; CPAP, continuous positive airway pressure; OSA, obstructive sleep apnea.

### Treatment of hypertension in patients with atrial fibrillation

Normotensive atrial fibrillation (AF) patients have a 2-fold mortality rate, 5-fold stroke, and 3-fold risk of developing HF when compared to individuals with a normal sinus rhythm. Hypertension is an independent risk for stroke in AF by 1.8–2.0 fold when compared to normotensive AF [186], therefore, hypertension with AF is a high risk. The prevalence of hypertension in AF patients is 62.0% by the Joint National Committee 8 definition and 79.0% by the American College of Cardiology/American Heart Association 2017 definition [186]. The diagnosis of hypertension in AF patients is ≥130/80 mmHg and is the threshold for treatment to the target BP of 120–129/<80 mmHg. Because of beat-to-beat BP variability, the BP measurement in AF needs at least 3 readings of office BP by auscultatory sphygmomanometer (*Strength of Recommendation IIa, Quality of Evidence B*) or automated oscillatory BP device with satisfactory SBP, but DBP may be overestimated by 2.1 mmHg [187] (*Strength of Recommendation IIa, Quality of Evidence B*). Some automated BP devices have been validated to detect AF during BP measurement, which is helpful to detect asymptomatic AF with a sensitivity of 0.98 and a specificity of 0.92 [188]. Recommendations for prevention and treatment of AF in hypertensive patients [189–200] are summarized in Table 27.

**Table 27.** Recommendations for hypertensive patients with AF

Recommendations	Strength of recommendations	Quality of evidence
Early detection and treatment of hypertension in patients at risk for AF is recommended.	I	C
Antihypertensive treatment is recommended to reduce the risk of incident and recurrent AF. The target for treatment is the same as for the general hypertensive population.	I	A
All major antihypertensive drug classes and combinations should be prescribed to control BP.	I	A
ACEis or ARBs and beta-blockers can be considered to prevent recurrent of AF.	IIa	B
At least 3 office BP measurements by auscultation can be recommended in AF to account for BP variability.	IIa	B
Automated oscillatory BP devices is an alternative for satisfactory SBP and modestly overestimated DBP measurement.	IIa	B
Beta-blockers are the preferred drug for heart rate control to be <110 bpm and targeting to <80 bpm in symptomatic patients.	I	B
Beta-blockers should not be combined with non-DHP CCBs.	III	C
Anticoagulants for stroke prevention can be considered in AF with BP ≥140/90 mmHg	IIa	B
If SBP is >160 mmHg, it is recommended to firstly reduce BP before initiation of anticoagulant in order to reduce risk of major bleeding and intracranial hemorrhage.	I	B
In AF patients with hypertension receiving anticoagulant, the treatment target and choice of agents are recommended the same as general hypertensive population.	I	B
Non-DHP CCBs for rate control should be used with caution because of drug interaction with oral anticoagulants and increased bleeding risk.	III	B

1 ACEis, angiotensin converting enzyme inhibitors; AF, atrial fibrillation; ARB, angiotensin receptor blockers; BP, blood pressure; DBP, diastolic blood pressure; DHP-CCB, dihydropyridine calcium channel blockers; SBP, systolic blood pressure.

## Hypertensive disorders in pregnancy

Hypertensive disorders in pregnancy constitute one of the leading causes of maternal and perinatal mortality worldwide. It has been estimated that preeclampsia complicates 2.0%–8.0% of pregnancies globally. In Asia, especially those in low- and middle-income countries including Thailand, hypertensive disorders are responsible for 9.0%–16.0% of maternal deaths [201, 202]. Preeclampsia is diagnosed when there is high BP with either proteinuria or end-organ involvement, all of which are of new onset and without other underlying explanatory conditions. The presence of end-organ involvement indicates a severe feature of preeclampsia. Diagnostic criteria of preeclampsia are shown in Table 28 [203].

**Table 28.** Diagnostic criteria of preeclampsia-eclampsia

Recommendations	Strength of recommendations	Quality of evidence
**High BP**		
After 20 weeks of gestation, pregnant woman with a previously normal BP, who has SBP ≥140 mm Hg and/or DBP ≥90 mm Hg on two occasions at least 4 h apart should be diagnosed as having high BP.	I	A
In a pregnant woman of any gestational weeks with unknown previous BP, SBP ≥160 mm Hg and/or DBP ≥110 mm Hg measured on two occasions within minutes (short interval), should be diagnosed as having high BP, to facilitate timely antihypertensive therapy for hypertensive crisis.	I	A
**Proteinuria**		
Quantitative protein leakage ≥300 mg/24-h urine collection (or this amount extrapolated from other collection methods) should be diagnosed as proteinuria.	I	A
Quantitative urine protein/creatinine ratio ≥0.3 should be diagnosed as proteinuria.	I	A
Qualitative (only when quantitative methods are not immediately available), urine dipstick reading ≥2+ should be diagnosed as proteinuria.	I	A
**End-organ involvement**		
Thrombocytopenia: platelet count <100,000/μL	I	A
Renal insufficiency: serum creatinine >1.1 mg/dL or doubling from the previous serum creatinine.	I	A
Impaired liver function: elevated serum liver transaminases to twice-normal concentration.	I	A
Pulmonary edema	I	A
Headache unresponsive to medication	I	A
Visual disturbances	I	A
Convulsion; generalized tonic-clonic seizure	I	A

1 BP, blood pressure; DBP, diastolic blood pressure; SBP, systolic blood pressure.

Maternal and fetal consequences need to be weighed while managing preeclampsia. Delivery is the only cure for preeclampsia, but the gestational age threshold may vary according to the presence or absence of severe features. Preeclampsia with end-organ involvement requires parenteral administration of magnesium sulfate to prevent convulsions. Preeclampsia with hypertensive crisis requires a timely hypertensive therapy. Choices of emergent antihypertensive therapies during pregnancy are shown in Table 29 [204–206].

**Table 29.** Antihypertensive agents used for emergent BP control during pregnancy

Recommendations	Strength of recommendations	Quality of evidence
Hydralazine 5 mg IV or IM bolus, then – IV boluses 5–10 mg every 20-40 min to a maximum cumulative dosage of 20 mg or – IV infusion of 0.5–10 mg/h	I	B
Nifedipine 10–20 mg orally, repeat in 20 min if needed, then – Oral 10–20 mg every 2–6 h to a maximum daily dose of 180 mg	I	B
Labetalol 10–20 mg IV bolus, then – IV boluses 20–80 mg every 10–30 min to a maximum cumulative dosage of 300 mg or – IV infusion of 1–2 mg/min	I	B

1 BP, blood pressure.

Women with risk factors for preeclampsia (as shown in Table 30) should receive low-dose (81 mg/d) aspirin for PE prophylaxis, initiated between 12 weeks and 28 weeks of gestation (optimally before 16 weeks of gestation) and continuing until delivery [207].

**Table 30.** Aspirin prophylaxis and level of clinical risk for preeclampsia-eclampsia

Recommendations	Strength of recommendations	Quality of evidence
Low dose aspirin is recommended in the presence of ≥1 of the followings – History of preeclampsia, especially with adverse outcomes – Multifetal gestation – Chronic hypertension – Type 1 or 2 diabetes – Renal disease Autoimmune disease	I	A
Low dose aspirin is recommended in the presence of ≥2 of the followings – Nulliparity – Obesity (BMI >30 kg/m^2^) – Family (mother or sister) history of preeclampsia – Unfavorable sociodemographic characteristics – Age ≥35 years Personal history factors (low birth weight, previous adverse pregnancy outcomes, at least 10 years pregnancy interval)	I	A
Low dose aspirin is not recommended – Previous uncomplicated full-term delivery	III	A

1 BMI, body mass index.

## Isolated diastolic hypertension

By the definition of SBP of <140 mmHg and DBP of ≥90 mmHg, the prevalence of isolated diastolic hypertension (IDH) defined by office BP in the general population is approximately 3.2%–5.2% [208, 209]. IDH is common in patients under 50 years old or 55 years old [208, 209] and is the most frequent hypertensive phenotype in individuals younger than 50 years. Overweight and obesity are highly associated with this hypertension phenotype [209]. The significant CV risks of IDH were confirmed in the systematic review and meta-analysis of 13 cohort studies (489,814 participants) [210]. Furthermore, age, especially <50 years, modified the effect of the association between IDH defined by both office and out-of-office BP and adverse CV outcomes [210, 211] (Table 31).

**Table 31.** Recommendations for management of IDH

Recommendations	Strength of recommendations	Quality of evidence
Lifestyle modification is recommended for all patients with IDH.	I	C
Recommendations for pharmacological interventions in general hypertensive patients should be applied to patients with IDH.	I	C

1 IDH, isolated diastolic hypertension.

A large-scale retrospective study of 71,297 participants with IDH demonstrated the protective effect of DBP reduction on CVD [212]. Unfortunately, there is currently no RCT indicating the benefit of treatment using antihypertensive medication. Although reduction of DBP is mainly focused on the treatment of IDH, excessively low SBP from the treatment should be of concern.

## Hypertension in the elderly and isolated systolic hypertension

Elderly people can be divided into 2 age groups: the early elderly group (60–79 years) and the very elderly group (80 years and above). There are changes in physical activities, functionality, psychological health, and lifestyle, including comorbidities, which affect treatment strategies, tolerability, and prognosis differently in those 2 groups of hypertensive persons (Table 32).

**Table 32.** Recommendations for management of hypertension in the elderly and ISH

Recommendations	Strength of recommendations	Quality of evidence
**60–79 years old individuals**		
The office threshold for drug treatment is ≥140/90 mmHg.	I	A
The primary target BP is SBP 130–140 mmHg and DBP 70–79 mmHg.	I	A
The secondary target BP is SBP 120–129 mmHg and DBP 70–79 mmHg if well tolerated to treatment.	I	B
**Individuals ≥80 years old**		
The frailty/functionality assessment should be done before initiation of treatment and repeated annually for monitoring.	I	C
The office SBP threshold for initiation of drug treatment is 160 mmHg	I	A
A lower SBP threshold in the 140–159 mmHg range may be considered in some selected persons.	IIb	C
The target office SBP 140–150 mmHg and DBP <80 mmHg is recommended.	I	A
The optional target SBP 130–139 mmHg may be considered if well tolerated and DBP is not too low.	IIb	B
In older persons, treatment should start with lower doses and titrate up slowly.	I	C
The search for orthostatic hypotension should be done systematically.	I	C
Do not aim to target office SBP <120 mmHg or DBP <70 mmHg.	III	C
Medication reduction or discontinuation can be considered in very elderly persons with a low SBP <120 mmHg or severe orthostatic hypotension or a high frailty level.	IIa	C
The treatment should be individualized in persons with moderate to severe level of frailty/functionality and/or dementia.	I	C
**ISH in the elderly persons**		
Due to SBP variability, repeatedly averaged office BP and HBPM may help in the diagnosis of ISH.	I	C
The primary target office SBP is 140–150 mmHg.	I	A
A reduction of office SBP target to 130–139 mmHg may be considered if well tolerated, and DBP is not too low.	IIb	B
CCBs and thiazide/thiazide-like diuretics are the drugs of choice. However, all other major drug classes can be used for compelling indications and combination therapy.	I	A
Initiation of 2-drug combination therapy is recommended in most ISH in elderly persons who are not frail.	I	C

1 BP, blood pressure; CCB, calcium-channel blockers; DBP, diastolic blood pressure; HBPM, home blood pressure measurement; ISH, isolated systolic hypertension; SBP, systolic blood pressure.

### The early elderly

The threshold office BP to consider treatment is the same as the younger age group (*Strength of Recommendation I, Quality of Evidence A*), and there are 2 target BP levels to reach sequentially.

Primary target: 130–139/70–79 mmHg (*Strength of Recommendation I, Quality of Evidence A*)

Secondary target: 120–129/70–79 mmHg, if treatment is well tolerated (*Strength of Recommendation I, Quality of Evidence B*)

No attempt should be made to reduce BP <120/70 mmHg (*Strength of Recommendation III, Quality of Evidence C*).

The physical activity recommendations are adjusted according to functional limitations for individual persons. The low salt diet or weight reduction may be applied cautiously, as it may cause loss of appetite or sarcopenia. There are more side effects of drug treatment in elderly persons, so “start low, go slowly” is a good strategy [213]. The choice of antihypertensive drugs can be any of the 5 major classes. However, beta-blockers can be used if there are compelling indications. The screening for orthostatic hypotension should be scheduled systematically (*Strength of Recommendation I, Quality of Evidence C*) as it is frequently found in elderly persons taking antihypertensive drugs. HBPM and ABPM are helpful to verify antihypertensive-related hypotension, postprandial hypotension, which can be aggravated by diuretics and vasodilators. ABPM can detect non-dipper, reverse dipper, and early morning surge phenotypes, and is useful for dose and timing adjustment of antihypertensive drugs in the elderly.

### The very elderly group

The same strategies, “start low, go slowly,” should be applied (*Strength of Recommendation I, Quality of Evidence C*). The beneficial effects of BP-lowering intervention are evident in the very elderly persons who are of good functionality to moderately frail [214, 215] but not in very frail individuals. In this group, frailty and functionality should be assessed before initiation of treatment and repeated annually to monitor the evolution of individuals’ status (*Strength of Recommendation I, Quality of Evidence C*).

BP threshold for initiation of drug treatment is 160 mmHg SBP (*Strength of Recommendation I, Quality of Evidence* *A*). The threshold of 140–159 mmHg may be considered in selected cases (*Strength of Recommendation IIb, Quality of Evidence C*) with target SBP of 140–150 mmHg and DBP <80 mmHg (*Strength of Recommendation I, Quality of Evidence A*). An optional target SBP of 130–139 mmHg, if well tolerated to treatment, is advised, although cautiously in individuals with DBP <70 mmHg (*Strength of Recommendation IIb, Quality of Evidence B*).

The reduction of treatment can be considered in persons ≥80 years with SBP <120 mmHg or severe orthostatic hypotension, or high frailty level (*Strength of Recommendation IIa, Quality of Evidence C*).

### Frailty and functionality assessment

The mental functionality can be assessed by using the minimental state examination (MMSE)-Thai 2002 from the Thai Ministry of Public Health. The physical limitation and dependency in daily life activities can be evaluated by Katz index from 0 (completely dependent) to 6 (completely autonomous) in bathing, dressing, toileting, transferring, feeding, and continence. For each daily activity, “0” means the person is unable to do it without assistance, 0.5 needs some assistance, and 1 means no need for any assistance. The MMSE score, Katz score, and other comorbidities are used to classify the level of frailty into 3 groups.

Group 1: mild frailty (relatively fit) has ≥5/6 Katz score, no dementia, MMSE >20/30, and can perform routine walking activities.

Group 2: moderate frailty has a profile between groups 1 and 3.

Group 3: severe frailty has a Katz score of <2/6, severe dementia or MMSE <10/30, or chronic bedridden or end-of-life conditions.

The treatment should be individualized in persons with moderate to severe levels of frailty/functionality and/or dementia *(Strength of Recommendation I, Quality of Evidence C*).

### Isolated systolic hypertension in the elderly

The definition of isolated systolic hypertension (ISH) is SBP ≥140 mmHg and DBP <90 mmHg. ISH is commonly found in persons >50 years of age, especially >70 years, women, and overweight individuals [216]. The meta-analysis suggested that treatment of ISH could reduce CVD outcomes by 26.0%, CV mortality by 18.0%, and all-cause mortality by 13.0% [217]. Because of BP variability in the elderly, HBPM and repeatedly averaged office BP may help in the diagnosis of ISH (*Strength of Recommendation I, quality of evidence C*).

The threshold BP to initiation of pharmacological treatment in ISH is ≥140 mmHg of SBP with consideration of functionality, frailty, and comorbidity. The primary target of SBP is 140–150 mmHg (*Strength of Recommendation I, Quality of Evidence A*), and the secondary SBP target is 130–139 mmHg, if well tolerated to treatment and DBP is not <70 mmHg (*Strength of Recommendation IIb, Quality of Evidence B*) [214].

ISH is difficult to treat, and a balance should be made between SBP reduction without too low DBP, causing organ hypoperfusion [218]. The drugs of choice are CCBs and thiazide/thiazide-like diuretics (*Strength of Recommendation I, Quality of Evidence A*), while ACEis and ARBs have lesser efficacy. Free or fixed combination therapy with ACEis or ARBs and CCBs or thiazide/thiazide-like diuretics should also be considered, particularly when compelling indications such as CAD, CKD, diabetes, and congestive HF coexist (*Strength of Recommendation I, Quality of Evidence B*). We recommend starting with dual combination therapy for older people with ISH who are not too frail (*Strength of Recommendation I, Quality of Evidence C*).

## Secondary hypertension

Secondary hypertension is not common but is possibly curable. Secondary hypertension should be suspected in patients aged <40 years who have resistant hypertension or who have an acute rise in BP. History, physical examination, and initial laboratory tests can provide clues to the causes of secondary hypertension. Causes of secondary hypertension are as in Table 33.

**Table 33.** Etiology of secondary hypertension based on organ systems

Endocrine causes	Primary aldosteronism Pheochromocytoma paraganglioma Cushing syndrome Hyperthyroidism and hypothyroidism Hypercalcemia and primary hyperparathyroidism Congenital adrenal hyperplasia Acromegaly
Metabolic disease	**OSA**
Renal causes	Renal parenchymal disease, e.g., glomerulonephritis, polycystic kidney disease, and CKD Renal artery stenosis
CV causes	Coarctation of the aorta

1 CKD, chronic kidney disease; CV, cardiovascular; OSA, obstructive sleep apnea.

## Resistant and refractory hypertension

**Figure 4.**
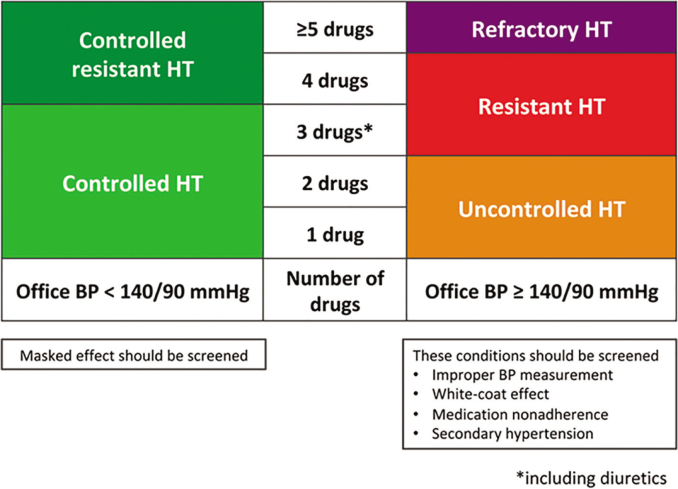
Classification of hypertension according to office BP control and number of antihypertensive drugs. BP, blood pressure.

**Figure 5.**
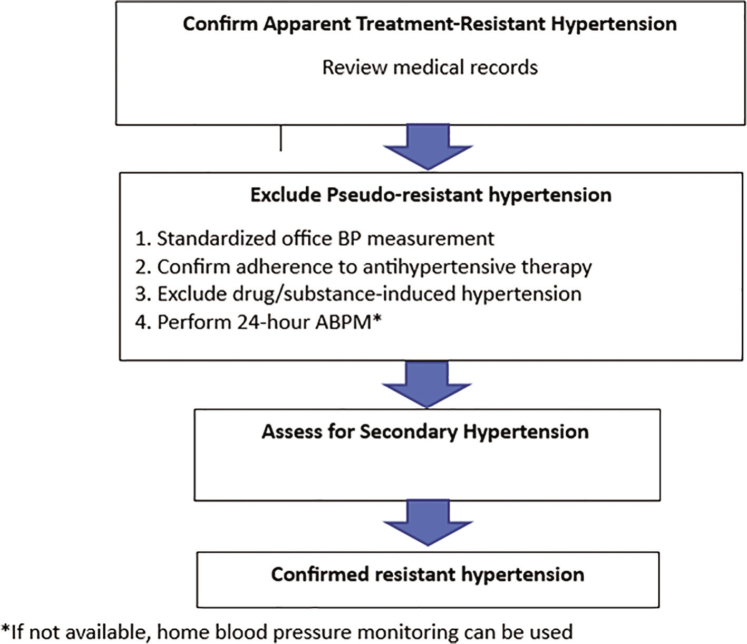
Algorithm suggested for the evaluation of resistant hypertension. ABPM, ambulatory blood pressure measurement; BP, blood pressure.

Resistant hypertension is high BP in a hypertensive patient that remains above goal despite the use of ≥3 antihypertensive agents of different classes, typically including a long-acting CCB, RAS blocker, either ACEI or ARB, and a diuretic, given at maximal or maximally tolerated doses [219, 220]. Refractory hypertension is BP that remains uncontrolled on maximal or near-maximal therapy, which is the use of at least 5 antihypertensive agents of different classes, including a long-acting thiazide-like diuretic and spironolactone [221]. Classification of hypertension according to office BP control and the number of antihypertensive medications is shown in Figure 4. The algorithm suggested for the evaluation of resistant hypertension is shown in Figure 5.

### Management of resistant hypertension

**Figure 6.**
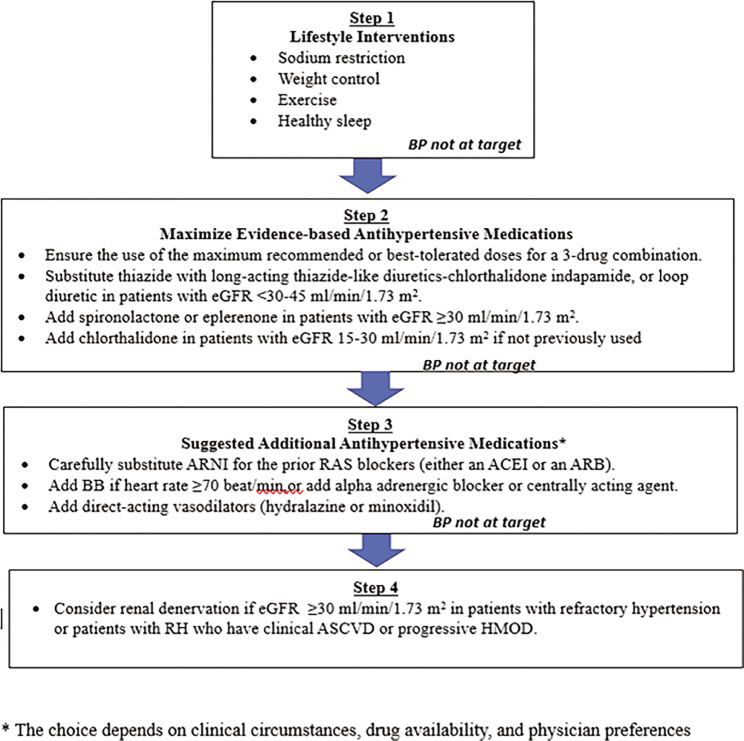
Recommendations for the management of resistant hypertension. ACEI, angiotensin converting enzyme inhibitors; ARB, angiotensin receptor blockers; ARNI, angiotensin receptor-neprilysin inhibitor; ASCVD, atherosclerotic cardiovascular disease; BP, blood pressure; eGFR, glomerular filtration rate estimation; HMOD, hypertension-mediated organ damage; RAS, renin angiotensin system.

Effective treatment of resistant hypertension requires life-style modification, along with a stepwise approach using antihypertensive medications with different mechanisms of action and considering device-based treatment when necessary. A suggested algorithm for managing resistant hypertension is shown in Figure 6.

### Device-based treatment

A few patients with resistant hypertension still have uncontrolled hypertension despite using full medication and having good adherence. Some of these patients may develop adverse reactions to various BP-lowering drugs, and the medications cannot be titrated to the required maximum doses. In these scenarios, consideration of device-based treatment is necessary. The device-based treatment that has been widely confirmed to be effective in resistant hypertension is catheter-based renal denervation therapy (RDN). To date, many clinical trials have proven that RDN can lower BP in patients with resistant and even refractory hypertension. However, the BP reduction after RDN can vary among patients. Since RDN is an invasive and costly treatment, we suggest using RDN only after careful and appropriate patient selection, such as refractory hypertension (*Strength of Recommendation I, Quality of Evidence C*) or resistant hypertension with established clinical ASCVD or progressive HMOD (*Strength of Recommendation IIa, Quality of Evidence B*) [222].

## Hypertensive emergency

A hypertensive emergency is defined as a critical clinical scenario where BP rapidly escalates beyond grade 3 hypertension thresholds. This acute elevation poses a substantial risk of ongoing organ damage, demanding rapid and effective medical actions to prevent further clinical deterioration [129, 130, 223]. It is crucial to note that the velocity of BP elevation is as critical as the absolute level; thus, in certain instances, BP may not reach the predefined thresholds yet still constitute a hypertensive emergency due to the rapidity of the increase and evidence of organ damage. This condition underscores the need for immediate therapeutic intervention to halt the progression of organ damage.

### Pre-critical symptomatic hypertension

To guide management strategies, this guideline introduces the term “pre-critical symptomatic hypertension (PSH)” as a replacement for “hypertensive urgency.” The traditional terminology lacks specificity regarding symptoms or clinical severity, often leads to confusion, and may result in inappropriate practices, such as overly rapid BP reduction using intravenous antihypertensives.

Distinguishing between a hypertensive emergency and PSH is crucial. PSH, defined as a significant and acute elevation in BP (often exceeding grade 3 hypertension), may present with symptoms such as mild headaches, dizziness, lightheadedness, or nausea, without evidence of organ damage [224].

### Evaluation for the presence of end-organ damage

Management strategies should encompass comprehensive evaluation for the presence of organ damage [223] (*Strength of Recommendation I, Quality of Evidence B*), including (1) CV system: acute coronary syndromes, HF, and aortic dissection, (2) renal system, namely, AKI, (3) central nervous system: hypertensive encephalopathy, stroke (both ischemic and hemorrhagic), and TIA, or malignant hypertension, (4) ocular changes: retinopathy, including hemorrhages and papilledema, and (5) other clinical presentations such as hypertensive thrombotic microangiopathy, which causes severe BP elevation associated with hemolysis, and thrombocytopenia in the absence of other causes and improvement with lowering BP.

The evaluation of acute organ damage involves a thorough physical examination as well as a series of diagnostic tests, including but not limited to creatinine, sodium, potassium levels, complete blood count, urine analysis, point of care ultrasonography, electrocardiogram, chest X-ray, fundoscopy, cardiac troponin levels, transthoracic echocardiogram, computed tomography angiography of the thorax or abdomen, and computerized tomographic or magnetic resonance imaging of the brain, as needed [223].

### Treatment of hypertensive emergencies

It is crucial to initiate treatment without delay (*Strength of Recommendation I, Quality of Evidence A*) to reduce the risk of further organ damage, morbidity, and mortality. The acute phase treatment in patients with hypertensive emergencies based on organ damage is shown in Table 34 [223, 225–227]. Evaluation and treatment of PSH and hypertensive emergency are summarized in Table 35.

**Table 34.** The acute phase treatment in patients with hypertensive emergencies based on organ damage

End-organ damage	Target BP (mmHg)	Time frame	Recommended treatment	Note
Acute coronary syndromes	SBP <140	Immediate	Nitroglycerine, labetalol*	Avoid over-BP reduction, SBP <110 mm Hg, or DBP <60 mm Hg.
Acute decompensated heart failure (cardiogenic pulmonary edema)	SBP <140	Immediate	Nitroglycerine, nitroprusside	Add on loop diuretics as needed.
Malignant hypertension	Decrease MAP by 20%–25%	A few hours	Nicardipine, labetalol	Nitroprusside can be used as a second line treatment.
Acute ischemic stroke	Adhere to established guidelines for acute stroke management.			
Acute hemorrhagic stroke				
Hypertensive encephalopathy	Decrease MAP by 20%–25%	Immediate	Nicardipine, labetalol	Nitroprusside can be used as a second line treatment.
Acute aortic diseases	SBP <120, and HR <60 bpm	Immediate	Esmolol, labetalol, nicardipine, and nitroglycerine	Combination therapy of 2 antihy-pertensive classes may be needed to control both SBP and HR.

1 BPM indicates beats per minute; DBP, diastolic BP; HR, heart rate; MAP, mean arterial pressure; SBP, systolic BP

* Avoid administering labetalol in instances of severe pulmonary congestion.

**Table 35.** Recommendations for the evaluation and management of patients presenting with hypertensive crises

Recommendations	Strength of recommendations	Quality of evidence
**General recommendations**		
Ensure the absence of acute organ damage through comprehensive physical examinations and necessary 1 laboratory tests. Assess for conditions such as ischemic and hemorrhagic stroke, acute HF, acute coronary 1 syndromes, acute aortic syndromes, and acute renal failure.	I	B
**Hypertensive emergencies**		
It is critical to reduce BP immediately to prevent further organ damage.	I	A
Intravenous agents are preferred for initial management due to their rapid onset of action and ease of titration. The choice of agent may vary based on the type of organ damage present.	I	A
Continuous monitoring of BP and reassessment of organ function and damage is crucial to guide ongoing 1 management and adjust treatment as necessary during the initial treatment phase.	I	C
**PSH**		
Identify triggers for accelerated BP increases, such as stress, anxiety, drug-induced hypertension, severe pain, withdrawal from antihypertensive medications, untreated hypertension, or severe white coat hyper-1 tension.	I	B
For individuals experiencing stress or anxiety, rest, breathing training, and anxiolytic medications can help alleviate symptoms and may assist in lowering BP.	IIb	B
For patients with moderate to severe pain, administer appropriate pain relief medication to improve symptoms and potentially aid in lowering BP.	IIa	B
Physicians should aim to lower BP gradually within 24–48 h instead of rapidly reducing BP to normal levels.	I	B
Upon discharge, ensure patients have been prescribed home medications.	I	B
For untreated hypertension, initiate antihypertensive therapy as per general treatment guidelines.	I	A
Schedule follow-up visits within 2–4 weeks for clinical evaluation and antihypertensive dose adjustments 1 to achieve target BP goals.	I	C
Patients discharged from the emergency department with PSH should be advised to subsequently monitor office or out-of-office BP measurements.	IIa	C

1 BP, blood pressure; HF, heart failure; PSH, pre-critical symptomatic hypertension.

## Reducing risk in hypertensive patients

The primary goal of treating high BP is to mitigate damage to vital organs and prevent the development of atherosclerosis. Since hypertension is a significant risk factor for atherosclerosis, individuals with high BP are more susceptible to CAD and cerebrovascular disorders. A holistic approach entails managing BP along with other CV risk factors, such as smoking, dyslipidemia, and DM (Table 36).

**Table 36.** Recommendations for reducing risk in hypertensive patients

Recommendations	Strength of recommendations	Quality of evidence
Patients should receive a risk assessment using Thai CV risk score.	I	C
Patients with ≥3 risk factors† should receive statin.	I	A
Patients who smoke should be advised or prescribed medication to quit smoking.	I	A
Patients with a calculated Thai CV risk score ≥10% (using blood results) can be considered to receive statin.	IIa	C
Hypertensive patients should avoid exposure to pollution, e.g., PM_2.5^•^_.	IIa	C
Aspirin should not be routinely used as primary prevention for every hypertensive patient.	III	A

† Risk factors consist of males, over 55 years of age, smoking, left ventricular hypertrophy, a history of premature CVD in the family, albuminuria, diabetes, atherosclerosis, or total cholesterol/HDL-C ≥6.

1 CV, cardiovascular; CVD, cardiovascular disease.

Numerous studies have demonstrated the efficacy of statins in reducing CV events in hypertensive patients. For instance, the Anglo-Scandinavian Cardiac Outcomes Trial-Lipid Lowering Arm (ASCOT-LLA) trial [228] revealed that atorvastatin 10 mg is more effective than a placebo for the primary prevention of CAD in hypertensive patients with CV risk factors. Recent research [229] further underscores the benefits of primary prevention with statins in hypertensive patients, leading to a 17.0% lower risk of all-cause mortality, a 15.0% reduction in CV mortality, and, in women, a 34.0% reduced risk of MI.

The use of aspirin in secondary prevention for established CAD and ischemic stroke is well established. However, the use of aspirin for primary prevention remains a subject of debate. A meta-analysis of 13 trials [230] involving 164,225 participants without a history of CVD found that aspirin use reduced the risk of CV events (absolute risk reduction, 0.41%) but increased the risk of major bleeding (absolute risk increase, 0.47%). Consequently, we do not recommend the routine use of aspirin for primary prevention (*Strength of Recommendation III, Quality of Evidence A*).

Recent studies from China [231] have shown a clear association between air pollution and elevated BP. Researchers estimated that 11.75% of hypertension cases in their study population could be attributed to ambient PM_2.5_ pollution. These findings indicate that prolonged exposure to PM_2.5_ pollution constitutes a significant risk factor for hypertension, particularly in the adult population in China. Other studies [232, 233] have also linked long-term exposure to PM_2.5_ with higher BP and an increased risk of hypertension prevalence.

In line with the American Heart Association’s scientific statement [234], there is a strong argument for offering the public practical, personalized strategies to reduce the potential health impacts of PM pollution. These strategies should adhere to 3 fundamental principles: (a) they should be practical, safe, and affordable; (b) they should be tailored to the risk profile of the individual, especially among susceptible populations; and (c) they should be accessible to anyone in need. Consequently, we recommend that hypertensive patients minimize their exposure to pollution, including PM_2.5_, even though there may be no specific clinical trials to support this recommendation.

## Telemedicine for BP control

Telemedicine has emerged as a valuable tool in managing chronic diseases like hypertension, offering innovative solutions to monitor and control BP effectively [235–238].

Home BP telemonitoring involves the remote monitoring and transmission of vital signs to medical centers utilizing digital HBPM devices. This approach enables healthcare providers to receive real-time data, allowing for timely intervention and personalized care. Several studies suggest that these interventions are associated with greater BP reduction and even reduced CV outcomes.

However, at present, it is premature to conclude the benefits of this virtual management approach at a national level since effective management requires dedicated healthcare personnel to interact with patients, adequate internet infrastructure and connectivity, and compliance with stringent privacy regulations due to security and privacy concerns. Of these, active collaborations from multiple stakeholders, such as the National Health Security Office, Ministry of Public Health, and Ministry of Digital Economy and Society, are vitally important to ensure its sustainability.

## Strategies to improve hypertension treatment and control

### The crucial problems

The major problems in hypertension control in Thailand are the increasing prevalence in the adult population and the inadequacy of awareness, treatment, and control, all of which have become worse in recent years [2].

The low level of awareness in Thailand can be explained not by the lack of systemic screening, but by the lack of confidence in the diagnosis of this mostly asymptomatic disease. The lack of confidence among patients results from the improper BP measurement technique used in the screening process, leading to unacceptable variations in BP. This discrepancy becomes apparent when patients discover that the screened BP significantly differs from previous or later measurements. Poor patients’ confidence is also a result of the inadequacy of time that healthcare personnel can spend explaining and answering many important questions that patients like to know. The inertia in diagnosis from healthcare personnel, especially the physician who should be the responsible person, is also very important. Unpublished data from many hospitals in Thailand showed that there was no proper diagnosis of hypertension, even when the in-hospital BP measurement was beyond grade 2. If there is still no general agreement in making a diagnosis, even among physicians, then there will be no trust among patients, who will be very confused about the diagnosis.

The lack of proper hypertension treatment in Thailand persists even years after the Thai government has provided full treatment coverage for almost the entire Thai population. The overpopulation of clinics can partly explain this in almost every government-supported health center. The long waiting time, the neglected feelings after very short attention by the physician, never having enough time to explain and ask about any possible adverse reaction from the treatment, and prescribing that remains the same almost every visit, whatever the BP is, are among the main factors that discouraged many patients from regular follow-up.

Despite numerous clinical studies suggesting benefits from starting treatment with antihypertensive medication in the form of a SPC [48], no antihypertensive medication in this form has been approved for use in the Thai National Essential Drug List.

### The suggested solutions

The higher prevalence of hypertension in the Thai adult population is caused by high salt intake, an increase in overweight and obesity, and air pollution. Trying to control all these risk factors systematically is not easy and will not be addressed here (Table 37).

**Table 37.** Recommendations to improve hypertension treatment and control

Recommendations	Strength of recommendations	Quality of evidence
Always use standardized BP measurements both in the diagnosis of hypertension and during follow-up.	I	A
Provide enough time for explaining and answering questions from the patients, especially when the first diagnosis of hypertension is made.	I	C
Use HBPM whenever possible.	I	B
HBPM is recommended to prevent inertia in the starting and adjustment of antihypertensive medication.	I	B

1 BP, blood pressure; HBPM, home blood pressure measurement.

From many observational cohort studies among Asian populations, individuals with BP 130–139/80–89 mmHg have a higher CV risk than those with a BP <120/80 mmHg [239–241]. This office BP category was previously classified as “high-normal” in the 2012, 2015, and 2019 Thai Hypertension Guidelines. The term “high-normal” creates the perception that this BP category is benign and requires no special attention. In this new guideline, we reclassify this BP category as “BP at risk.” Hopefully, this new classification will increase awareness among both patients and healthcare workers in Thailand.

A low level of awareness is partly due to a lack of confidence in hypertension diagnosis. The suggested strategies that we recommend in this guideline are:

1. Always use standardized BP measurement both in diagnosis and follow-up to guarantee the precision and quality of diagnosis (*Strength of Recommendation I, Quality of Evidence A*).
2. Provide enough time for explaining and answering questions from the patients, especially when the first diagnosis is made (*Strength of Recommendation I, Quality of Evidence C*).
3. Use HBPM (*Strength of Recommendation I, Quality of Evidence B*).

The most important steps to limit doctors’ inertia in making diagnoses and in starting and adjusting antihypertensive medications are to improve knowledge about the increased CV risk associated with hypertension and the protective effects of adequate BP control. This goal needs the wide dissemination of treatment guidelines. Patient knowledge and empowerment, as well as HBPM use, are also important for providing appropriate and timely feedback to the doctor. We recommend using HBPM to prevent inertia in starting and adjusting proper antihypertensive medication (*Strength of Recommendation I, Quality of Evidence B*) [242]. The possibility of a more widely used tele-transmission of home BP measurement can be helpful, but it is still not widely available in Thailand at this time. The advent of technology has now allowed the use of telehealth and health-related mobile applications that can be installed on smartphones. These interventions can improve patients’ education and lead to greater BP reduction; however, at present, it is premature to conclude on the benefits of these approaches in general. This new system can be useful but requires more responsible healthcare personnel to guarantee its effectiveness. Due to the limited manpower in most government-supported NCD clinics, the application of this system may be slow and will be available only in some clinics.

Other considerations include more frequent patient visits, a simple hospital treatment protocol, and the allowance of medication adjustments from nurse specialists or pharmacists under a doctor’s supervision. There was a proposal to give incentives to treatment units or doctors based on the number of patients achieving good BP control. The suggestion of combining 3 approaches, which are (a) measure BP accurately, (b) act rapidly, and (c) partner with the patients, has been shown to improve BP control and reduce therapeutic inertia in a large group of hypertensive patients [243].

Therapeutic inertia can also occur from patients’ reluctance and refusal to increase the number of doses of the medications. Changing the medication to an SPC with fewer pills per day, even with a larger dose, and allowing time for a good discussion with the patients can solve these problems in most cases.

A patient-centered approach is the foundation of the care plan for patients with hypertension. The patient empowerment process will enable patients to access the correct information and actively participate in their medical decisions. This process offers a better chance to improve adherence to medications and lifestyle modification [238, 244, 245]. Patient empowerment can be useful in sharing decisions in goal setting, provision of feedback channels, self-monitoring, education, and motivation, which the use of telemedicine and mobile health technologies can assist. Successful providerpatient interaction needs a team-based approach with professionals and a multidisciplinary team of health providers. All these approaches need to be personalized under careful consideration of the cultural, economic, and social variables of each patient.
